# The role of the sewer system in estimating urban emissions of chemicals of emerging concern

**DOI:** 10.1007/s11157-022-09638-9

**Published:** 2022-10-23

**Authors:** Caterina Zillien, Leo Posthuma, Erwin Roex, Ad Ragas

**Affiliations:** 1 Department of Environmental Science, Radboud University, Nijmegen, The Netherlands; 2 Centre for Sustainability, Environment and Health, National Institute for Public Health and the Environment (RIVM), Bilthoven, The Netherlands; 3 Centre for Zoonoses and Environmental Microbiology, National Institute for Public Health and the Environment (RIVM), Bilthoven, The Netherlands

**Keywords:** Micro-pollutants, Wastewater, Environmental fate, Biodegradation, Modeling

## Abstract

The use of chemicals by society has resulted in calls for more effective control of their emissions. Many of these chemicals are poorly characterized because of lacking data on their use, environmental fate and toxicity, as well as lacking detection techniques. These compounds are sometimes referred to as contaminants of emerging concern (CECs). Urban areas are an important source of CECs, where these are typically first collected in sewer systems and then discharged into the environment after being treated in a wastewater treatment plant. A combination of emission estimation techniques and environmental fate models can support the early identification and management of CEC-related environmental problems. However, scientific insight in the processes driving the fate of CECs in sewer systems is limited and scattered. Biotransformation, sorption and ion-trapping can decrease CEC loads, whereas enzymatic deconjugation of conjugated metabolites can increase CEC loads as metabolites are back-transformed into their parent respective compounds. These fate processes need to be considered when estimating CEC emissions. This literature review collates the fragmented knowledge and data on in-sewer fate of CECs to develop practical guidelines for water managers on how to deal with in-sewer fate of CECs and highlights future research needs. It was assessed to what extent empirical data is in-line with text-book knowledge and integrated sewer modelling approaches. Experimental half-lives (*n* = 277) of 96 organic CECs were collected from literature. The findings of this literature review can be used to support environmental modelling efforts and to optimize monitoring campaigns, including field studies in the context of wastewater-based epidemiology.

**Graphical abstract:**

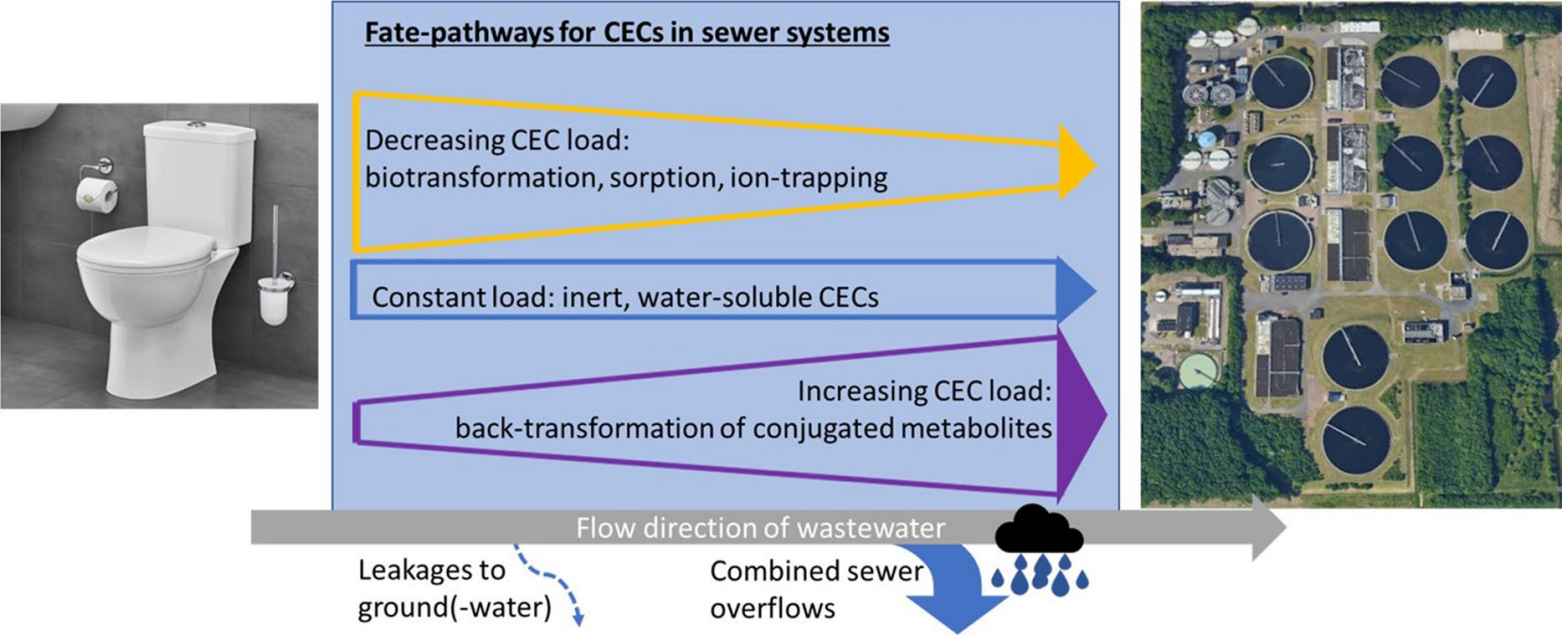

**Supplementary Information:**

The online version contains supplementary material available at 10.1007/s11157-022-09638-9.

## Introduction

Water quality and sanitation are key challenges of the twenty-first century as reflected in the United Nations Sustainable Development Goal number 6 (United Nations n.d.). Given the increasing importance of wastewater surveillance in monitoring the spread of SARS-CoV-2 (e.g. Wade et al. 2022) and other infectious diseases, similar programs could be used to monitor otsearch aimingher water-related pollution entering the environment via treated or untreated wastewater. While wastewater treatment plants (WWTPs) effectively remove nutrients from wastewater, conventional treatment steps often fail to remove complex chemical structures such as pharmaceuticals, surfactants or other chemicals of emerging concern (CECs). In high-income countries where wastewater collection and treatment is well-established, joint efforts from science and policy aim to improve surface water quality with regards to CECs (Dijksma 2017; European Commission 2010). Initiatives to limit the use of CECs and to implement advanced treatment technologies could help to reduce the exposure of wildlife and ecosystems to the chemical cocktail used by our modern societies (Finckh et al. 2022).

Many studies underline the prominent role of WWTP effluents in the emission of CECs to the environment. Examples include the review of Bunting et al. (2021) reporting relatively high groundwater concentrations of CECs in the neighbourhood of WWTPs, and the study of Ben Mordechay et al. (2021) who found CECs in concentrations ranging from ng/g to μg/g in almost all sampled fruits and vegetables grown with reclaimed WWTP effluents in Israel. Sousa et al. (2018) reported traces of commonly used pharmaceuticals in nearly all sorts of waters, from rivers to lakes and groundwater bodies worldwide. Wilkinson et al. (2022) showed the presence of CECs in the environment is a global issue, with surface water concentrations of pharmaceuticals, particularly of analgesics and antibiotics, in low- and middle-income countries exceeding those reported for high-income countries by up to five orders or magnitude.

The societal need to map and control CECs is currently being hampered by a lack of detailed insight into the emissions of-, exposure to- and eventual effects of CECs. The continuous analytical advances of the past years, particularly in non-target screening, enable faster and more efficient detection of CECs in the environment. While large amounts of empirical data on exposure and effects of CECs are currently being gathered, the immense number and variety of CECs used in society and industry calls for a more efficient and systematic approach to tackle this issue. To illustrate, measuring all 23,000 substances registered under the European regulation REACH ("Registration, Evaluation, Authorisation and Restriction of Chemicals"; ECHA, n.d.) and the > 1400 active pharmaceutical ingredients for human consumption registered within the European Union (EU; European Medicines Agency, n.d.) is both financially and technically unfeasible. Furthermore, the diversity of compound properties challenges existing sampling techniques as well as current methods for analysis. Additionally, limited availability of conclusive toxicity data hampers holistic effect assessment, particularly of unintended mixtures. Therefore, we need to complement analytical efforts with innovative modelling approaches to reliably predict the fate and effects of CECs based on parameters such as production and usage data, chemical characteristics, environmental conditions and species characteristics. Developing such predictive tools (e.g. Douziech et al. 2017; Gustavsson et al. 2022; Li et al. 2021) will expand our capabilities to manage CECs, e.g. by anticipating potential problems before they arise and by illustrating the impact of alternative interventions.

To reliably estimate wastewater-related CEC emissions from urban areas to the environment, it is important to consider both mass of CECs emitted into sewers and the fate processes occurring in the sewer system. Several studies indicate that certain compounds present in wastewater are already partially degraded in the sewer system before reaching the WWTP (e.g. Choi et al. (2020); McCall et al. (2016a); O’Brien et al. (2017). However, detailed information on the fate of CECs in sewers is fragmented. This review aims to collate that information in order to answer the questions “What is the role of the sewer system in estimating wastewater-related CEC emissions from urban areas to the environment?” and “What fate processes should be considered in emission estimations and how can these processes be modelled?”.

**Fig. 1:**
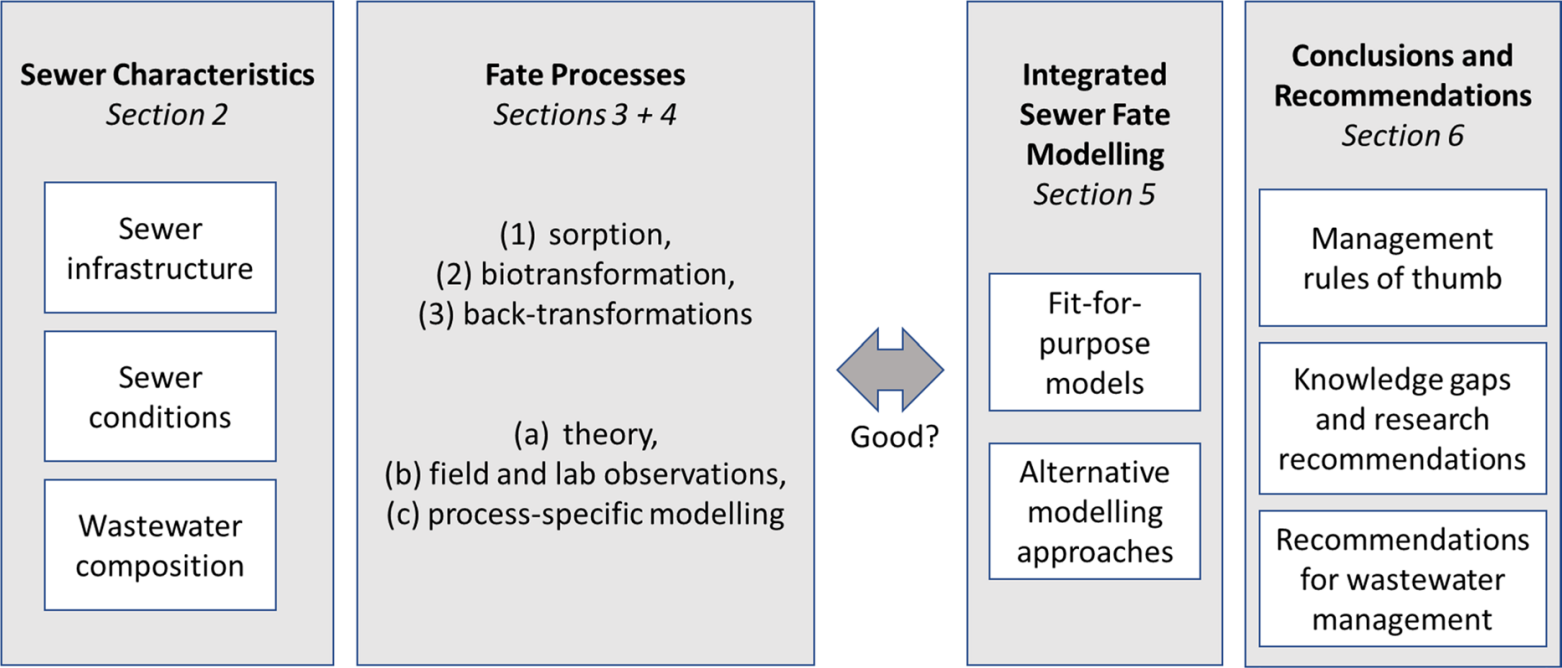
Conceptualization of the literature review

To limit the scope of this study, this review focuses on organic CECs that occur in low concentrations in the environment like pharmaceuticals, surfactants and illicit drugs. As many forms and concepts of sewer systems and wastewater treatment exist around the world, in this review we refer to sewers as an underground piping network, typically made from concrete, brick or PVC to convey wastewater from various origins (domestic, industrial, stormwater run-off) in urban settings to the closest WWTP. The time it takes for wastewater to reach the WWTP from any source within the sewer catchment is referred to as sewer residence time. Underway, physical and biochemical fate processes can affect the load of CECs upon passage of the sewer system. The theoretical representation of sewer systems, wastewater hydrology, CEC emissions and fate processes in numerical modelling approaches are assessed in integrated fate models. The conceptualization of this review is illustrated in Fig. 1.

This literature review is structured along four main sections. Section 2 characterizes sewer systems in terms of infrastructure, wastewater composition and microbial community to identify which system-related parameters are important when assessing the fate of CECs in sewers. Section 3 discusses the most important fate processes that affect the load of CECs in wastewater. For each process, we briefly describe the underlying principles, then discuss how each process can be modelled and contrast this with empirical data as reported in the scientific literature. Section 4 reports the results of analyzing a set of collated empirical data on CEC fate in experimental and field studies. Differences between experimental set-ups as well as their implications for resulting half-lives and their application in fate modelling are highlighted. Section 5 summarizes the integrated sewer models that have been described in the literature, discusses their common features and limitations, and assesses whether these models are fit-for-purpose. Each section ends with a brief summary of key findings in relation to the research aim of this study and relevant knowledge basis for the subsequent analyses. Finally, the main findings of this literature review are synthesized in Sect. 6. We present some pragmatic rules of thumb for water professionals to decide for what compounds and under which circumstances in-sewer fate needs to be considered when estimating CEC emissions to WWTPs. For the research community, we highlight current knowledge gaps and end with a brief outlook on opportunities for future research.

## Sewers and their characteristics

The fate of CECs in sewers is influenced by compound properties and the characteristics of the sewer system. For soluble CECs, the residence time of wastewater in the sewer system is a crucial parameter as it indicates the time window in which biochemical processes can take place. CECs that can undergo sorption to biofilms or sewer sediment, could remain longer in the sewer system as wastewater. The effect of sorption on the fate of CECs is discussed in Sect. 3.1. Section 2 focuses on the link between sewer infrastructure and residence time of wastewater Sect. (2.1), CECs present in sewers Sect. (2.2), and the prevailing environmental conditions Sect. (2.3).

### Sewer infrastructure and residence time

Sewers transport wastewater from the emission sources towards the WWTP by gravity, force or a combination thereof (Ort et al. 2014). Free-flow gravity systems (GS) are usually designed to meet a minimum flow velocity of 0.6–0.9 m per second to prevent sedimentation and the production of hydrogen sulfide (Hvitved-Jacobsen et al. 2013; Kapo et al. 2017). Since gravity sewers often convey a mix of sanitary wastewater and storm water run-off, the residence time can vary substantially depending on the intensity and duration of the rainfall and the resulting sewer discharge. In pressurized systems, also called rising mains (RM), the flow velocity is controlled by pumping stations and their respective capacity. As described e.g. in Jelic et al. (2015), pressurized systems operate with storage basins that activate the pump once a certain water level is reached.

The sewer residence time of wastewater can vary considerably between sewer catchments but also within single sewer systems as the flow of wastewater is highly variable. The mean sewer residence time of wastewater in 29 European cities ranges from less than an hour till up to 12 h with an average of about 5 h, depending on the size and layout of the sewer system (Ort et al. 2014). McCall et al. (2017) found that the diurnal flow for less populated upstream areas of a catchment is more variable than for downstream collection pipes. The authors quantified daily temporal variability of sewer residence times in large catchments to be + 20% to − 10% (McCall et al. 2017). Atinkpahoun et al. (2018) assessed the effect of commuting on wastewater dynamics in Nancy (France) and found that collective behavior like morning routines, lunch breaks and dinner preparations were clearly visible in the WWTP inflow. When modelling in-sewer fate, it is important to consider parameters that affect the sewer residence time like sewer layout and mode of transportation of the system. Depending on the research question, factors that drive variability in flow patterns like rainfall intensity or population habits regarding water usage need to be considered.

In combined sewer systems, periodic and incidental events of variable wastewater flow can cause substantial problems for water managers and the environment. Especially during summer months, extended periods of low discharge can lead to longer sewer residence times that in combination with temperatures > 15 °C cause oxygen depletion (Hvitved-Jacobsen et al. 2013). This favors the formation of hydrogen sulfide resulting in sewer smell and increased deterioration of concrete sewer pipes (Hvitved-Jacobsen et al. 2013; Vollertsen et al. 2015). In contrast, severe rain events can lead to shorter sewer residence times and can cause sewer systems to overflow if water volumes exceed the storage capacity of the system. Consequently, untreated wastewater enters the environment leading to short-term pollution of surface waters. For instance, Munro et al. (2019) report that the combined sewer system of London (England) overflows weekly even following low intensity rain events, leading to 39 million tons of untreated wastewater entering the Thames river in an average year. The ecological effect of combined sewer overflows (CSOs) can be detrimental to the quality of receiving waters. Launay et al. (2016) for example studied the effect of CSOs on receiving water bodies in a small catchment in Southern Germany. After CSO events, surface water concentrations of some CECs exceeded their European environmental quality standards even though wastewater was simultaneously diluted by the storm and surface water. Given that CSOs can be an important source of CECs to the environment, not only the emission route via WWTPs need to be considered when assessing CEC emissions from urban areas but also CSOs.

Environmental issues can also arise when sanitary wastewater and storm water are being collected separately. In sewer systems where storm water is collected via a separate network of pipes and overground ditches, the sanitary wastewater is not being diluted and can be conveyed to the WWTP via smaller pipes. Surface run-off is typically stored in overground ponds or channels, infiltration sites (e.g., wadi’s), or discharges of storm water directly into surface waters. In the Western world, separated sewer systems are often built in regions where heavy rainfalls occur regularly as well as in newly built development areas. However, due to the more complex network, misconnected pipes can cause cross-contamination between both systems. For the Netherlands, it has been estimated that up to 5% of the sewer pipes is connected erroneously, leading to continuous emission of untreated wastewater into the environment (Bollmann et al. 2019). Besides, it is becoming increasingly clear that untreated surface run-off represents a relatively constant source of pollution directly affecting local surface water quality (Peter et al. 2020), especially when the storm water network is not connected to a WWTP. In terms of estimating CECs emissions to the environment, local characteristics of sewer systems and rainfall patterns can be important and should be considered.

Sewer pipes are no closed-off systems and can therefore interact with shallow groundwater or bank infiltrate. Since most urban sewer pipes are made of concrete or sometimes brick, the material itself allows water to permeate into the sewers (groundwater infiltration, e.g. Heiderscheidt et al. 2022) or from the sewers to the surroundings (exfiltration, e.g. Wolf et al. 2012). When estimating CEC loads in wastewater, particularly exfiltration could be a relevant emission pathway potentially affecting mass balances. Besides corrosion of sewer pipes, e.g. due to hydrogen sulfide, external factors like land subsidence, invading tree roots or heavy traffic can damage sewer pipes leading to sewer leakages particularly when infrastructure is aged and groundwater tables are low (Nguyen et al. 2021). While extensive research efforts focused on quantifying the extent of sewer leakages and resulting exfiltration rates, the only agreeable common seems to be that sewer leakages are highly variable and strongly dependent on local circumstances and the chosen modelling approach (Rutsch et al. 2008). Nguyen and Venohr (2021) report substantial damages in public and private sewers in Germany and estimated 60 defects per km of sewer pipe as a national average based on various data sources including sewer inspection reports. While consensus exists that sewer exfiltration mainly affects the direct surroundings of leaking sewers (Wolf et al. 2012), there is an ongoing debate about the potential environmental and human health impacts of the resulting groundwater contamination. Since exfiltration rates can vary substantially between cities and across seasons (Nguyen et al. 2021), more research is needed to assess whether emission estimations of wastewater-related CECs could be affected.

### Wastewater composition and CECs

The composition of wastewater is strongly influenced by local characteristics and activities (Atinkpahoun et al. 2018). Domestic wastewater is typically rich in nutrients (kitchen wastewater, toilet) and contains various CECs like fragrances, surfactants used in personal care products (via shower and bath; e.g. Camacho-Muñoz et al. (2014)), detergents (via laundry), disinfectants in cleaning products (via sink), intentionally ingested compounds like pharmaceuticals or illicit drugs, but also chemicals we are unintentionally exposed to like pesticides, plastic additives and flame retardants (via toilet and sink; e.g. González-Mariño et al. 2021). Scientific literature on the composition of industrial wastewater is limited and suggests that the composition might vary substantially according to the type of industry and potential on-site treatment (Camacho-Muñoz et al. 2014). Storm water run-off contains substantial amounts of various pollutants including polycyclic aromatic hydrocarbons (PAHs; e.g. Deffontis et al. 2013), heavy metals (e.g. Baralkiewicz et al. 2014), airborne contaminants including micro-plastics (e.g. Dris et al. 2015), traffic-related contaminants (e.g. Peter et al. 2020), but also CECs of diffuse sources like pesticides, polychlorinated biphenyls (PCBs; Langeveld et al. 2020) and poly-fluoroalkyl substances (PFAS; e.g. Codling et al. 2020).

Previously it was hypothesized that contaminants in storm water run-off would enter the environment mostly via the first-flush effect: contaminants accumulate on the surface during prolonged period of dry weather and being flushed in high concentrations within the first moments of a severe rain event. However, recent studies demonstrate that also shorter, less intense and more frequent rain events can contribute substantially to emission of traffic-related CECs like the corrosion inhibitor benzotriazole and tire-related compounds like hexa(methoxymethyl)melamine (Peter et al. 2020). Furthermore, Peter et al. (2020) postulate that especially traffic-related CECs ‘may exhibit transport-limited rather than mass-limited dynamics’ and therefore represent a continuous source of contaminants to the environment.

### Sewer microbes and wastewater temperature

Since most biochemical reactions are temperature dependent, variability in wastewater temperature within the sewer system needs to be considered when modelling the fate of CECs. Tchobanoglous et al. (2003), for instance, report annual average wastewater temperatures for the United States of America (US) between three and 27 °C depending on climatic conditions with a representative mean of 15.6 °C. Similarly, observed mean temperatures of WWTP influent in the dataset of Ort et al. (2014), ranged between 10 and 20 °C for 23 out of 28 European cities (Figure S5). Although collected data points are limited to March and April of two subsequent years, given the geographical spread of the participating cities, the annual mean wastewater temperatures for most temperate cities are likely to stay within the observed range of 5–28 °C overall. Relatively high observed maximum temperatures (28 °C) of WWTP influent in the two most southern cities (Cyprus) in April could indicate that in these latitudes, wastewater temperatures might even increase well beyond 30 °C during summer months. Schilperoort and Clemens (2009) assessed the temperature of combined wastewater over a two-kilometer residential sewer in the Netherlands in December 2008 and concluded that upstream temperatures close to household connections were more variable (up to 35 °C) than downstream temperatures (12–14 °C). This suggests that the composition of microbial communities in sewer systems could vary considerably not just among cities and across seasons as indicated by McLellan and Roguet (2019) but also within a single sewer catchment.

Sewer microbes occur in almost all sewer compartments: suspended in the wastewater itself, attached to sewer walls in the form of biofilms and also sewer sediment accommodates microorganisms (Hvitved-Jacobsen et al. 2013; McLellan and Roguet 2019). In general terms, mainly heterotrophic microorganisms thrive in sewers and can degrade wastewater constituents (Hvitved-Jacobsen et al. 2013; Tchobanoglous et al. 2003). As for most biological activity, also sewer microorganisms are temperature- and pH-dependent. With every 10 °C temperature increase, the activity rate more or less doubles (Hvitved-Jacobsen et al. 2013). The optimum temperature for wastewater microbes typically ranges between 25 and 30 °C at pH 6.5–7.5 (Tchobanoglous et al. 2003). The temperature dependence of biotransformation in wastewater is often described by the Arrhenius function (e.g. Hart and Halden 2020). However, a study conducted by Meynet et al. (2020) suggests that the classical Arrhenius function overestimates the biotransformation of CECs at wastewater temperatures above 20 °C. The authors attribute the observed misfit to changes in microbial community and its activity, and propose an adapted, quadratic Arrhenius-based function.

Two studies assessed the composition of the microbial community in US sewers and found that the human fecal microbiome accounted for only 15–20% of the encountered bacteria (McLellan and Roguet 2019; Newton et al. 2015). Most bacterial families residing in sewers, such as *Arcobacter*, *Acinetobacter*, *Aeromonas* and *Trichococcus*, are not of human origin (McLellan and Roguet 2019). Overall, microbial communities in sewers appeared stable and similar across 71 US cities (Newton et al. 2015). Variations in non-fecal microorganisms between cities seemed closely linked to a city’s temperature profile, while the variation in microorganisms of human origin was much less affected by changing temperatures. This suggests that biotransformation rates in sewers might vary more in temperate regions due to seasonal changes in temperature, than in regions with stable yearly temperatures. More research into the composition of microbial communities and their function in sewer systems is needed to confirm or reject this hypothesis.

Regarding microbial activity in sewers, especially biofilms received growing attention over the past years. Biofilms are slimy layers of bacteria that grow attached to the submerged parts of sewer walls. While biofilms in gravity sewers can grow several millimeters thick and are fluffy in their appearance, biofilms in pressure sewers are smoother and less thick due to higher flow velocities. McLellan and Roguet (2019) found that biofilms and sewer sediments often share the same bacterial strains, though at varying proportions. Some bacterial families like *Synergistacea* were enriched up to 16% in biofilms while other families were more abundant in wastewater or sewer sediments. *Arcobacter*, for example, was also found 10% more abundant in wastewater than in biofilm or sediments, suggesting that this genus occurs mainly in suspension. Generally, suspended biomass seems less efficient in degrading CECs than biofilms, especially under anaerobic conditions (e.g. Ramin et al. 2017).

### Recap: sewer characteristics in relation to CEC fate

Local circumstances regarding the infrastructure of the sewer system, climatic pattern and factors causing variability in wastewater flow need to be considered when estimating wastewater-related CEC emissions from urban areas to the environment. Wastewater temperatures affect the microbial community in the sewer and can therefore affect the fate of CECs. Variability in wastewater temperature across seasons and within sewer catchments might translate into variable biotransformation rates. Sewer residence time of wastewater is an important parameter for soluble CECs. Depending on the research question at hand, factors contributing to variability in sewer residence time need to be considered. Periodic events such as reduced flow during extended dry periods can increase sewer residence times. During these periods, defect sewer pipes could facilitate exfiltration of wastewater from sewer systems especially in areas with low groundwater tables. In contrast, rain events can cause substantially shorter sewer residence times due to increased sewer flow in combined systems and provoke sewers to overflow when storage capacity of the sewer system is exceeded. More research is needed to assess the magnitude of wastewater and CECs entering the environment via sewer leakages, CSOs and separated rainwater sewers, and their ecological effects. Furthermore, the composition and function of microbial communities in sewers, also in relation to the prevailing variability of environmental conditions, need to be studied in more detail.

## In-sewer fate processes: theory and modelling

The characteristic composition of wastewater in combination with the prevalent physicochemical conditions in sewers create a unique environment, offering a habitat to a multitude of microorganisms and enabling biochemical processes that can affect CECs on their journey through the sewer system. In this section we discuss the most important fate processes that affect the load of CECs in sewers. Whereas sorption Sect. (3.1) and biotransformation Sect. (3.2) typically lead to a decrease in dissolved CEC loads, back-transformation of conjugated metabolites Sect. (3.3) can result in increasing loads for selected compounds. For each process, we briefly describe the underlying principles, then discuss how each process can be modelled and contrast this with empirical data described in literature.

### Sorption, ionization and ion trapping

Besides an abundant microbial community, wastewater also contains substantial amounts of solid (organic) matter, either in suspension or settled in the form of sewer sediment. The particle size ranges from colloidal (0.001–1 µm; Hvitved-Jacobsen et al. 2013) to macro scale (e.g., leaves or toilet paper). Biomass and organic solids are generally negatively charged due to their organic carbon content. Sorption is governed by charge and therefore a reversible, abiotic process. For neutral compounds, sorption is driven by hydrophobicity (e.g., expressed as log K_d_) and consequently linked to intrinsic compound properties. Neutral compounds with high log K_d_ values, i.e. ≥ 2, can sorb onto deposited sewer sediments, suspended particles, suspended biomass, or biofilms growing on sewer walls (Carballa et al. 2008; Joss et al. 2006).

In environmental fate models, sorption of CECs is typically modelled as a function of compound properties and the fraction of organic carbon (f_OC_) present in the medium of interest. Crabtree 1989) in Hvitved-Jacobsen et al. 2013) proposed a classification scheme for sewer sediment including the fraction of organic carbon present in each category. According to this classification, organic carbon content ranges from 0.07 in coarse bed material, over 0.22 in fine solids (top layer) to 0.61 in biofilm. The wastewater treatment model SimpleTreat uses f_OC_ values of 0.3 for raw wastewater and 0.37 for activated sludge (Struijs 2014). For neutral compounds, the sorption coefficient K_d_ (Eq. 1) is derived either using the experimental K_OC_ value or by using quantitative structure–property relationships (QSPRs) based on the K_OW_ (Eq. 2) according to Sablijc et al. (1995).

1 $$K_{d} = K_{OC} * f_{OC}$$

2 $$K_{OC} = 1.26* {K_{OW}} ^{0.81}$$

Overall, sorption of neutral organic CECs to suspended colloidal solids in wastewater appears more important than to deposited sewer sediment. Hajj-Mohamad et al. (2017) attribute this increased sorption to suspended solids to the substantially higher organic carbon content in suspended as compared to settled sewer sediments: In a set of laboratory tests using real sewer sediment to assess the sorption behavior of CECs (i.e., three pharmaceuticals, a pharmaceutical metabolite and caffeine), all compounds showed higher concentrations in suspended solids (f_OC_ = 0.52) as compared to settled sediments (f_OC_ = 0.06). Similarly, Kaeseberg et al. (2018) used real sewer sediment in laboratory tests and found that the antibiotics they studied sorbed more to particle sizes smaller than 200 µm as compared to larger particles. Laboratory studies have shown that sorption of CECs to sediments (Li et al. 2020) or biofilm (Ramin et al. 2017) can lead to subsequent biotransformation.

Most studies agree that under laboratory settings, sorption to sewer sediments proceeds quickly, i.e. in the order of minutes (Hajj-Mohamad et al. 2017; Kaeseberg et al. 2018). Less consensus exists around the speed of desorption following changes in environmental conditions e.g. due to dilution with stormwater: for some compounds (i.e., caffeine and carbamazepine in Hajj-Mohamad et al. (2017) and all antibiotics in Kaeseberg et al. (2018)) quick desorption (minutes) was observed, while for other compounds desorption rates in the range of hours to days were reported (Hajj-Mohamad et al. 2017). This means that sorption of CECs in sewers depends mainly on compound properties (lipophilicity) and composition of wastewater (f_OC_).

Two studies question the suitability of classical sorption tests to inform the mechanistic understanding of sorption in wastewater. Hajj-Mohamad et al. (2017) noted differences in observed sorption behavior of neutral compounds in relation to mechanistic understanding based on the theoretic relationship between K_d_ and log K_OW_. While it was expected (based on log K_OW_ of − 0.07) that caffeine would sorb the least, experimentally it showed the highest K_d_ value of all studied compounds for suspended solids. According to the authors this suggests that octanol is a poor surrogate to describe sorption to the organic fraction present in wastewater. The study by Bagnis et al. (2018) provides a possible explanation reporting that “wastewater is mainly composed of proteinaceous material which binds organic contaminants more weakly than humic-like substances typical of freshwater”. Furthermore, Hajj-Mohamad et al. (2017) found that spiking sewer sediments with reference compounds can lead to substantially higher experimental sorption coefficients (K_d_) as compared to K_d_ values deducted from experiments at concentrations comparable to environmental levels. The shortcomings of traditional approaches to estimate sorption of CECs e.g. based on log K_OW_ underline the need for more empirical data and understanding when assessing the fate of CECs in sewers.

Sorption can also be an important fate process for ionic and ionizable compounds. The degree of ionization of a compound is determined by the type of compound (acid or base), the dissociation constant (pKa) of the compound and the environmental pH. The pH of wastewater is commonly in the range of 6.5–8, even though incidental pH values as low as 3.9 were reported for WWTP influent (Ort et al. 2014). At common pH values of 6.5–8, organic bases with pKa values of 8.5–10 would be present mainly as protonated cations. Protonated cations are positively charged and could undergo electrostatic sorption to negatively charged biomass or suspended organic matter. In contrast, organic acids with pKa values between 6.5 and 10 are mainly present as negatively charged anions at pH ranges typical for wastewater. Anions are unlikely to undergo sorption in wastewater as their negative electrostatic charge will repel them from negatively charged sewer particles (Bagnis et al. 2018). Since ionization is reversible for weak acids and bases, changes in pH could theoretically affect the sorption potential of ionized compounds. However, Gulde et al. (2014) did not observe any pH-dependent sorption trends when studying the removal of 15 cationic pharmaceuticals in the presence of activated sludge. Similarly, Bagnis et al. (2018) studied the sorption and desorption behavior of several CECs in synthetic wastewater upon dilution with surface water and found that at higher dilution factors (8–10) lipophilicity appeared to be a better indicator for observed sorption behavior of neutral and cationic compounds than their charge. One possible explanation is that ionized compounds, independently on whether positively or negatively charged, are more water-soluble and therefore more mobile than their neutral forms.

In a more recent study, Gulde et al. (2018) observed that ion trapping could contribute substantially to the removal of amines during wastewater treatment using activated sludge. Ion trapping refers to the diffusion of the neutral form of amines into the cells of eukaryotic organisms where the compound is ionized. This prevents diffusion back outside the cell leading to a ‘trapped ion’ within an eukaryote (Gulde et al. 2018). The authors conclude that the resulting decrease in dissolved concentrations have often been erroneously attributed to pH-dependent biotransformation (Gulde et al. 2018). While this process has so far only been studied under laboratory settings and using activated sludge, it seems possible that ion trapping could occur already in the sewer system. Given that many CECs including pharmaceuticals are ionizable (Droge and Goss 2013), ionization could play a substantial role in the fate of CECs in sewers.

When modelling the fate of CECs in sewers, the ionized fraction of a compound can be calculated as a function of the prevailing pH and the dissociation constant of a compound using the Henderson-Hasselbalch equation. Sorption of ionized compounds is typically modelled following the approach by Franco et al. (2013) (Eqs. 3 and 4) for monovalent acids (3) and monovalent bases with pKa ≤ 4 (4).

3 $$K_{OC, acid} = \varphi_{n} 10^{{0.54\log K_{OW} + 1.11}} + \varphi_{i} 10^{{0.11\log K_{OW} + 1.54}}$$

4 $$K_{OC, base} = 10^{{0.31\log D_{OW} + 2.78}}$$

where $\varphi_{n}$ is the fraction of the neutral species, $\varphi_{i}$ is the fraction of the ionized species as calculated using the Henderson-Hasselbalch equation with pH = environmental pH – 0.6. The pH dependent K_ow_ distribution coefficient log D_ow_ is calculated via Eq. 5 as described, e.g., in Lin et al. (2010).

5 $$\log D_{ow} = \log K_{ow} + \log \frac{1}{{1 + 10^{{\left( {pKa - pH} \right)}} }}$$

### Biotransformation

Given the high abundance of organic matter in combination with a variety of sewer microbes present in wastewater (Sect. 3.3), it is hardly surprising that biotransformation can be important in sewer systems. Biotransformation refers to the biologically induced transformation of chemical structures. As a result, compounds can be either degraded completely (mineralization) or partially to structurally similar compounds. Given that it remains unclear whether complete mineralization is actually achieved in sewers, we use the term ‘biotransformation’ throughout this review as it describes the biological transformation of CECs in our opinion more appropriately than the term ‘biodegradation’.

The mechanistic understanding of biotransformation is currently limited. Fate models consider biotransformation via first-order half-lives (DT_50_, Eq. 6) or rate constants (k_bio_), thus relying on empirical or predicted biotransformation rates to account for aerobic or anoxic biotransformation. Consequently, compound specific DT_50_s or transformation rates in water and sludge are required to compute meaningful results. However, this represents the biggest limitation in application of any fate model since empirical biotransformation data – especially for wastewater – is extremely scarce, even when including results from standardized OECD tests (Comber et al. 2019). When modelling in-sewer transformation of CECs, McCall et al. (2017) concluded that uncertainty due to the limited availability of biotransformation rates can be more important than uncertainty introduced by other model inputs.

6 $$DT_{50} = \frac{\ln \left( 2 \right)}{{k_{bio} }}_{ }$$

Wastewater-based epidemiology (WBE) studies generated a substantial number of empirical DT_50_s for CECs in wastewater over the past years. Since the inherent aim of WBE studies is typically to estimate consumption of mostly illicit drugs by a given population based on wastewater measurements, the compound selection of these studies is biased towards opioids and other drugs of abuse. As the water concentrations of many drugs of interest showed to decrease in time frames comparable to average sewer residence times, without correction for in-sewer fate, consumption estimations would be prone to underestimation.

The observed variability of DT_50_s for single compounds across studies suggests that biotransformation strongly depends on highly variable local conditions including microbial communities and/or sewer conditions. For example, anaerobic bioreactor DT_50_s for cocaine were reported ranging from 5.9 h (Li et al. 2018) to 35 h (Senta et al. 2014), and for methadone ranging from 1.1 h (Gao et al. 2017) to 160 h (Ramin et al. 2016). Smaller differences in DT_50_s were observed depending on the redox conditions prevailing during experiments. O’Brien et al. (2017) reported for iopromide DT_50_s of 10.7 (anaerobic) and 10 h (aerobic) and for codeine DT_50_s of 2.1 (anaerobic) and 3.8 h (aerobic). In general, the large variability in experimentally derived DT_50_s limit their application to assess fate of CECs in other circumstances.

### Back-transformation of metabolites to parent compounds

While most exposure and effect research focuses on parent compounds of pharmaceuticals, metabolites are rather underrepresented even though large shares of administered doses are not excreted as parent compound but as metabolites. For example, Sathishkumar et al. (2020) reviewed metabolic transformation processes of diclofenac in mammals and concluded that diclofenac undergoes direct conjugation, hydroxylation, conjugation following hydroxylation and hydroxymethoxylation upon ingestion. Osorio et al. (2014) reports that in humans diclofenac is metabolized in the liver resulting in the main metabolites 4′hydroxydiclofenac (30%), 4′-5dihydroxy diclofenac (15%), 1-O acyl glucuronide (15%) and 5-hydroxydiclofenac (10%). Only about 1% of orally administered diclofenac is excreted as parent compound (Vieno and Sillanpää 2014). Similarly, only 5% of orally administered acetaminophen is excreted as parent compound while 60–80% is excreted as acetaminophen-glucuronide and 15–35% as acetaminophen sulfate (CBG-MEB 2019). Not only pharmaceuticals but also unintentionally ingested plasticizers are reported to form conjugated metabolites which have been observed to deconjugate in wastewater under laboratory settings (He et al. 2021).

In terms of mass balancing, such conjugated metabolites are of particular interest as enzymatic hydrolysis (Osorio et al. 2014; Pérez and Barceló 2008) can lead to deconjugation and thus back-transformation to the parent compound. The enzyme β-glucuronidase, secreted by *E.coli* bacteria (D’Ascenzo et al. 2003) but also present in human urine (Wu et al. 1998; Zhang et al. 2020), for example, could contribute to deconjugation of glucuronides (Vieno and Sillanpää 2014). A study conducted by Lee et al. (2012) suggests that abiotic hydrolysis could also contribute to the deconjugation of glucuronide metabolites, though slower and to a lesser degree as compared to enzymatic hydrolysis. This finding, however, was not confirmed by Brown et al. (2020) who did not observe abiotic deconjugation but who highlighted that enzymatic deconjugation proceeds in the order of hours.

It has been hypothesized that deconjugation during wastewater treatment is responsible for negative removal efficiencies as reported for a number of CECs including carbamazepine (McCall et al. 2017), estrogens (D’Ascenzo et al. 2003), diclofenac (e.g., Stülten et al. 2008) and a number of antibiotics (e.g. Muriuki et al. 2020). Brown et al. (2020) showed under laboratory settings that solids load, aerobic microbial activity as well as retention time and compound properties were interlinked and together determine the course of conjugation and de-conjugation processes. The results of their study indicated that for example the antibiotic sulfamethoxazole was simultaneously degraded and back-transformed from the related glucuronide and acetyl metabolites, leading to only minimal removal during long retention times in the presence of microbial activity. A field study conducted by Jelic et al. (2015) suggests that de-conjugation also plays a role in real sewers as they observed the formation of sulfamethoxazole and irbesartan along a 7 km long pressure sewer pipe in Spain. Similarly, Gao et al. (2018) observed an increase of nicotine-related biomarkers in a pressurized sewer in Australia. Therefore, conjugation and de-conjugation are relevant processes in sewer systems and need to be taken into account when performing mass balances for CECs.

So far, only Delli Compagni et al. (2020) made an effort to incorporate back-transformation due to deconjugation into a sewer-specific fate model (IUWS_MP model) by specifying a simple, compound-specific first-order process rate. Modelling back-transformation this way again heavily relies on empirical data regarding deconjugation rate constants. Given that deconjugation of glucuronide metabolites is reported to proceed within several hours (Brown et al. 2020; D’Ascenzo et al. 2003) we assume that under most sewer residence times back-transformation is very likely to be initiated already upon passage through the sewer system and completed during wastewater treatment as observed by D’Ascenzo et al. (2003). However, when assuming full deconjugation of metabolites into their respective parent compound Delli Compagni et al. (2020) observed overestimation of parent compounds in WWTP influent. This suggests, that measured WWTP removal efficiencies might underestimate ‘true’ removal if only parent compounds are measured since conjugated metabolites likely back-transform into parent compounds while parent compounds are being removed. This could explain the reported misfit of predicted and observed WWTP removal efficiencies (e.g. Douziech et al. 2017).

### Recap: theory and modelling of fate processes in sewers

Sorption, ionization, ion trapping, biodegradation and back-transformation can all be relevant processes for the fate of CECs in sewers. Their relevance in a particular situation depends on factors such as CEC properties (e.g., solubility and degree of ionization), wastewater composition (e.g., organic matter content and microbes) and physical conditions (e.g., temperature and pH; Figures S4-6). The traditional approach to estimate sorption to organic matter based on log K_ow_ (often applied to soils and suspended matter) seems less suitable for wastewater due to its high biomass and protein content. Ion trapping could be important for the fate of ionizable compounds in sewers and WWTPs but requires more research. Biotransformation is likely the most important fate process for CECs in sewers. The observed variability of DT_50_s for single compounds across studies shows that degradation strongly depends on local conditions. This implies that experimentally obtained DT_50_s cannot be easily extrapolated to other conditions, underlining the need for better mechanistic understanding of the factors driving biodegradation. Furthermore, sorption and biotransformation in wastewater appear to be related given that laboratory studies have shown that sorption of CECs to sediments (Li et al. 2020) or biofilm (Ramin et al. 2017) can lead to subsequent biotransformation. This implies that the traditional concept of bioavailability (i.e., a sorbed molecule is unavailable for toxicity and transformation) might not apply to wastewater conditions. Back-transformation of conjugated metabolites can also be a relevant fate process. Under most sewer residence times, back-transformation is very likely to be initiated already upon passage through the sewer system and completed during wastewater treatment as enzymatic deconjugation is reported to proceed within hours particularly for glucuronide-conjugates (Brown et al. 2020; D’Ascenzo et al. 2003). This could explain the reported misfit of predicted and observed WWTP removal efficiencies (e.g. Douziech et al. 2017), but needs to be confirmed by additional research.

## Empirical sewer fate data

In the following sections, we summarize the results of an extensive literature search aiming to collate empirical data on the fate of CECs in sewer systems. Details on the search methodology, analyses performed and the results can be found in the SI1. We first present a general overview (4.1), followed by a presentation based on the experimental set-up of the study in order of increasing complexity: OECD tests (4.2), bioreactor studies (4.3), pilot studies Sect. (4.4) and field studies (4.5).

### General overview

**Fig. 2:**
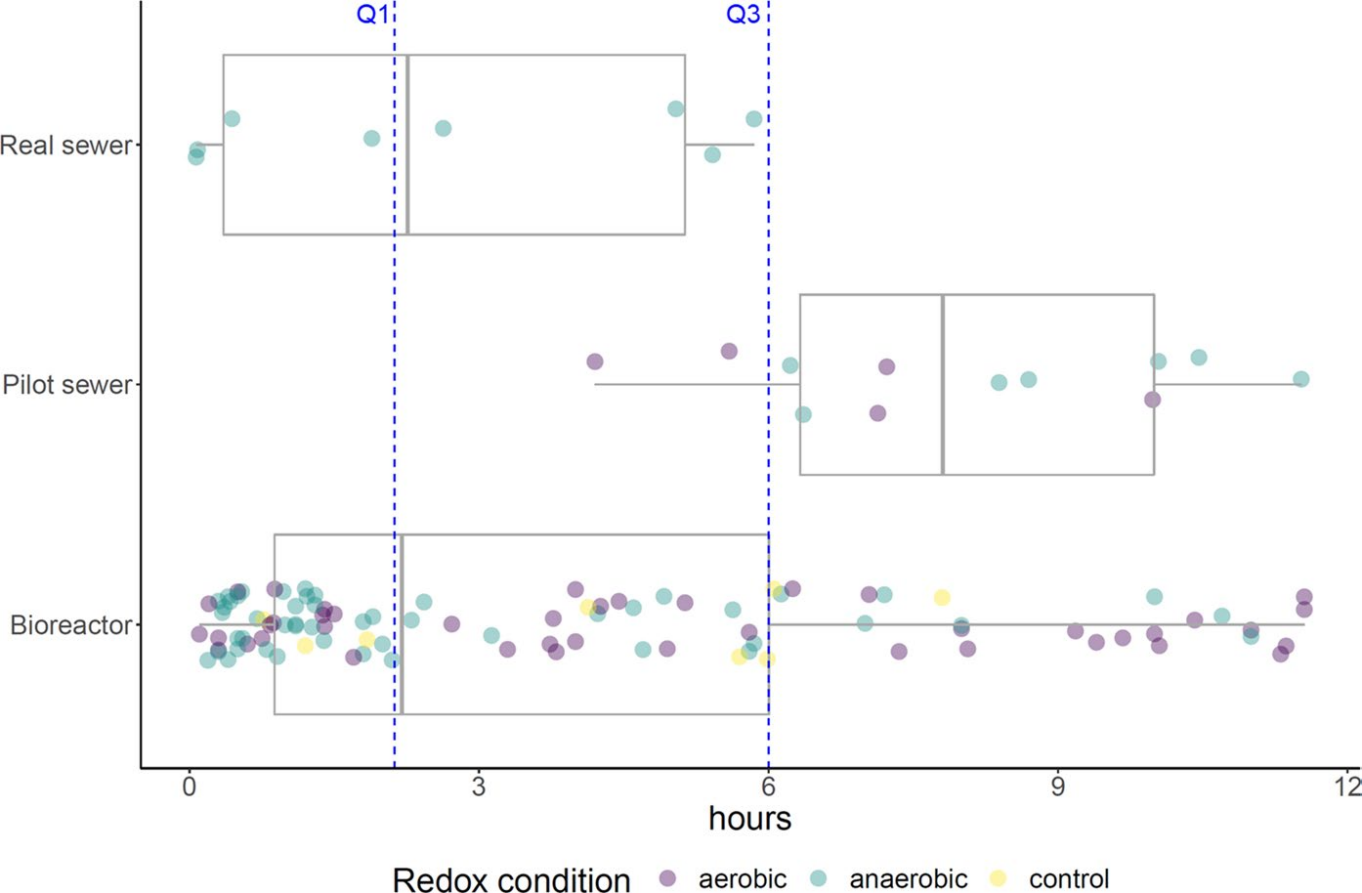
Reported DT_50_s (*n* = 120) shorter than 12 h for 55 CECs according to type of study and prevailing redox conditions. Blue dotted lines represent 1st and 3rd quartile of mean sewer residence time reported for 29 sewer catchments in Europe in Ort et al. (2014). The variation of the mean wastewater residence time (Sect. 2) is added for illustrative purposes

We collected in total 277 DT_50_s for 96 unique compounds from literature, listed in SI2. 120 DT_50_s of 55 unique compounds are shorter than 12 h and can thus be considered relevant for urban sewer catchments (Fig. 2). The vast majority of these DT_50_s were reported for bioreactor tests (100 of 120), while only few entries were reported for pilot and field studies (12 and 8 respectively). 53% of these DT_50_s were obtained under anaerobic conditions (*n* = 64), while 40% were obtained under aerobic conditions (*n* = 48). Some bioreactor experiments report DT_50_s obtained via abiotic control experiments which typically use autoclaved wastewater. 85 DT_50_s of 65 unique compounds are shorter than 6 h, i.e. the 3rd quartile of the mean sewer residence times reported for 29 European sewer catchments by Ort et al. (2014). This means that in 75% of those sewer system less than half of the input load of these 65 compounds would reach the WWTP inlet. Vice versa, estimating usage or consumption of these compounds from measurements at the WWTP inlet would lead to underestimations of more than factor 2.

### OECD test 314A

Standardized OECD tests assess the degradability of compounds in various environmental media. In 2008, a specific guideline to assess biotransformation of chemicals in wastewater was published (OECD 314). The guidelines describe five tests of which one (314A) specifically assesses biotransformation in sewer systems under low dissolved oxygen (DO) concentrations (0.2–1 mg/L) and for non-volatile compounds at environmentally relevant concentrations (OECD 2008). Our literature search identified only 1 publication applying the test to a number of surfactants in domestic wastewater (Menzies et al. 2017). We conclude that OECD test 314A is hardly performed in practice or the results are not being reported in the public scientific literature.

### Bioreactor tests

Our literature review resulted in 18 studies assessing fate of CECs using lab-scale bioreactors to simulate different sewer systems (Table 1). Bioreactor or batch tests are experiments conducted in vessels of different volumes ranging from a few 100 mL to several liters and often performed in the context of WBE. Generally, bioreactor studies have a similar set-up: A real wastewater sample is collected, divided into various subsamples which represent different sewer settings such as gravity sewers or pressurized sewers by adjusting the redox conditions and the biofilm area-to-volume (AV) ratios. To create sewer-like conditions in the reactors, most studies cultivate biofilms on reactor walls or plastic carriers for several weeks to years before starting the experiment (e.g. Ramin et al. 2017; Banks et al. 2018). Subsamples are spiked with a known concentration of a CEC mixture and monitored throughout the experiment. To deduct DT_50_s or biotransformation rates (k_bio_) from such experiments, the in-sample concentration of CECs is measured at several points in time to create a dissipation curve which is subsequently fitted to a kinetic model such as zero-order (linear decrease) or first-order (exponential decrease) model.

**Table 1:** Overview of bioreactor studies assessing the stability of CECs in wastewater

Reference	Compounds studied	Location	Wastewater used	Sampling method	Volume reactor	Sewer type	AV ratio	T ww	pH	HRT	Sampled matrix	Spiked?	Triplicate?	Duration of experiment	Remarks
					[L]		[m-1]	[°C]		[h]					
Banks et al. (2018)	Tobacco, alcohol	Australia	Real, domestic. Collected weekly, new sample for each replicate	n.a	0.75	RM, GS, CR	RM: 72.5;GS: 50	20	7.2	6	Water	Yes	Yes	12 h	Sewer biofilms have been cultivated for 8 years; pH and temperature comparable with OECD 314; new water sampler for each replicate
Brown et al. (2020)	Pharmaceuticals (conjugates + parent)	Canada	Real WWTP	24 h composite sample	3	n.a	n.a	n.a	6.5–8.4	2; 24	Water	Yes	Triplicate/ duplicate	24 h	Studied deconjugation; samples taken at WWTP: primary, primary activated, secondary effluent
Choi et al. (2020)	Pharmaceuticals, consumer products	Australia	Real, domestic	n.a	0.75	RM, GS, CR, BFF (biofilm-free)	GS: 50;RM: 72	23	7	12	Water	Yes	Yes	24 h	
Cormier et al. (2015)	Pharmaceuticals	Canada	Surface water, raw ww after primary sludge tank	Grab sample	0.03	oxic	n.a	4; 21	7.4–7.5	n.a	Water	Yes	Duplicate	130 days	OECD test 309; study duration too long to be representative for in-sewer degradation
Gallé et al. (2019)	Pharmaceuticals, consumer products	Luxembourg	Activated sludge	Grab sample (AS)	4	AS (WWTP)–GS		22	7	n.a	Water	Yes	Duplicate	7 h	
Gao et al. (2017)	Illicit drugs, opiates	Australia	Real, domestic. New sample for each batch	n.a	0.75	RM, GS, CR	RM:72.5; GS: 50	20	7.5	12	Water	Yes	Yes	12 h	
Li et al. (2018)	Illicit drugs, opiates	Australia	Real, domestic	Weekly grab sample	0.75	RM, CR	72.5	23	n.a	6	Water	Yes	Yes	12 h	
Li et al. (2020)	Illicit drugs, opiates	Australia	Real, domestic	n.a	3.2	GS	5.2; 10; 15; 20	22	7.2	n.a	Water	Yes, selectively	Yes	12 h	Sediment originally collected from GS, cultivated in reactor for 5 years, no re-suspension of sediment; Test 1: biomarker transformation; Test 2: Biomarker stability at varying pH. Sampled only water phase
Lin et al. (2021)	Pharmaceuticals	China	WWTP influent	n.a	1	RM, GS, CR, WW	60	at pH 7.4: 4; 15; 25; 35	at 25C: 2, 5, 7, 9	24	Water	Yes	Yes	72 h	Biofilm cultivated from activated sludge; sampling moments quite coarse; separate tests for hydrolysis, sorption, biodegradation and combined
McCall et al. (2016b)	Mainly illicit drugs	Switzerland	n.a	n.a	0.75; 1.5; 3	RM, GS	17; 33; 67	21	7–8	n.a	Water	Yes	n.a	24 h	Used real biofilms from 2 locations and lab biofilm grown with real ww
O’Brien et al. (2017)	Pharmaceuticals	Australia	Real, domestic	n.a	0.75	RM, GS, CR	70.9	20	7.5	6	Water	Yes, selectively	Yes	12 h	Test conditions meet OECD 314
O’Brien et al. (2019)	Oxidative stress biomarkers	Australia	Real, domestic	n.a	0.75	RM, GS, CR	RM: 72.5; GS: 50	n.a	7.5	n.a	Water	Yes	Yes	12 h	
Ramin et al. (2016)	Illicit drugs, metabolites	Denmark	WWTP influent	Grab sample (ww and primary sludge)	7; 4	Aerobic, anaerobic	n.a	14–15	8–9	n.a	Water	Yes	n.a	48 h	Biotransformation (raw ww), sorption (diluted sludge), abiotic (mineral water)
Ramin et al. (2017)	Illicit drugs, metabolites	Denmark	Biotransformation test (BT): pre-clarified ww, centrifuged, vacuum filtered. Sorption test (SO): WWTP effluent, vacuum filtered	Grab sample	0.961	BT: Aerobic, anaerobic; SO using suspended biofilm)	175	BT:17 SO: 15	BT: 8.7–9.2; SO:7.9	n.a	Water	Yes	No	BT: 48 h, SO: 4 h	Reactors fed continuously (4L/d) during 7 months in the dark
Senta et al. (2014)	Illicit drugs, metabolites, opiates	Croatia	Real, secondary effluent WWTP	24 h composite	0.3	n.a	n.a	10, 20	2, 7.5	n.a	Water	Yes	No, only analysis in duplicate	72 h	Significantly longer DT_50_s for winter (10 °C) conditions. Not clear if reactors were supplied with O_2_. Study also assessed impact of in-sewer degradation for various residence times
Thai et al. (2014)	Illicit drugs	Australia	Real, domestic	n.a	0.75	RM, GS, CR	RM: 72.5; GS: 65.4	20	7.5	6	Water	Yes	Yes	12 h	
Thai et al. (2019)	Natural hormones	Australia	Real, domestic	Weekly grab sample	0.75	RM, GS, CR	n.a.; probably same as Thai et al. 2014	20	7	6	Water	Yes	Yes	12 h	
van Nuijs et al. (2012)	Illicit drugs, metabolites	Belgium	WWTP influent	5L pooled influent ww from 7 WWTPs in Belgium	0.75	n.a	n.a	20	7.5	26	Water	Yes	Duplicate	26 h	Different environmental set-up: stability in ww assessed (no GS or RM conditions), samples were hand-shaken, CR refer to not-spiked. 50 mL of each batch were withdrawn and frozen for later analysis

*RM*, rising main (pressurized sewer); *GS*, gravity sewer; *CR*, control experiment (typically wastewater without biofilms); *AV ratio*, biofilm area to sewer volume ratio [m^2^/m^3^]; *AS*, activated sludge; *T ww*, temperature in wastewater; *ww*, wastewater; *HRT*, hydraulic retention time; *n.a.*, data not available in article/supplementary information

Notably, half of the reviewed bioreactor studies were performed by the same research group in Australia, using the same or very similar experimental set-ups (including the same biofilm growing in bioreactors) as well as the same source of wastewater. Furthermore, most studies assessed the fate of compounds at neutral pH levels of 7–8.5 and water temperatures of around 20 °C; only Lin et al. (2021) assessed a wider spectrum of pHs (pH 2, 5, 7, 9) and temperatures (4, 15, 25, 35 °C). All studies used real wastewater collected from neighboring sewer systems or WWTPs to fill the bioreactors. Compounds of interest were in all cases spiked (unless background concentrations were high enough to exceed the detection limits) and measured several times over the duration of the experiment. Under aerobic conditions, 50% of all DT_50_s (*n* = 117) best fitted first-order kinetics, 38% fitted zero-order kinetics and for 12% of the reported DT_50_s second-order models resulted in best fit. Also, under anaerobic conditions, first-order kinetics represented most DT_50_s (62%) while zero- and second-order models fitted fewer DT_50_s (25% and 13%, respectively; *n* = 98). In contrast, DT_50_s derived from abiotic controls (*n *= 33) fitted zero-order models better (58%) than first-order models (42%).

Noteworthy, all reviewed studies only measured the dissolved fraction of compounds and attributed compound loss in the water phase to degradation. However, it remains unclear whether disappearance was the result of primary or full transformation as none of the studies – probably due to very high costs – used radio-labelled compounds which would allow to make this distinction. Similarly, the effect of sorption on the observed dissipation of compounds in bioreactor settings is difficult to determine. Theoretically it is possible to create circumstances within a bioreactor that could allow to distinguish between biotic and abiotic processes which in reality occur simultaneously. For example, Lin et al. (2021) autoclaved wastewater before spiking the compounds of interest to study the effect of abiotic processes without the interference of active biomass. Fine-tuning of reactor settings in such ways is laborious but could help to better understand underlying mechanisms of fate processes.

Some studies acclimated the bioreactors for several months or years to promote biofilm growth on the reactor walls comparable to real sewers (e.g. Gao et al. 2019). Most studies used raw wastewater to grow and maintain biofilms: one study collected real biofilm from a gravity sewer (McCall et al. 2016b), another study grew biofilm from activated sludge sampled from a WWTP (Lin et al. 2021). Some studies inoculated plastic carriers with biofilms to study the effect of the ratio between biofilm area and sewer volume (AV ratio; McCall et al. 2016b; Thai et al. 2014). Other studies employ ‘control reactors’ without biofilms to study the effect of suspended biomass compared to attached biomass (biofilms). In order to compare the effects of the different conditions, it would be necessary to normalize derived DT_50_s based on the amount of biomass used in each experiment. However, microbial analysis, e.g., via 16S rRNA, is hardly ever performed, leaving the AV ratio as the only reported measure of active biomass from which only the surface area of the biofilm but not necessarily its thickness can be calculated. Similarly, information on the composition of microbial communities is hardly assessed or reported, even though a potential link between specific microbial communities and the large variability in biotransformation rates observed for single compounds, is widely accepted (e.g. Douziech et al. 2018; Nolte and Ragas 2017). This limits the comparison of reported DT_50_s between studies.

When simulating gravity sewers at laboratory scale, bioreactor tests likely overestimate degradation as aeration and continuous stirring result in better mixing and higher oxygen levels as compared to real sewers. Dissolved oxygen concentrations in real gravity sewers showed to decrease exponentially within two km even at low water temperatures (Huisman et al. 2004). Also the ratio of water depth to pipe diameter (also termed y/D ratio) showed to have a significant impact on the depletion of dissolved oxygen (DO) as the reaeration decreases with increasing water depth. Hvitved-Jacobsen et al. (2013) demonstrated without additional aeration, DO concentrations decrease sharply from around 4 g O_2_/m^3^ to complete depletion at a water temperature of 20 °C, after 2.5 km of length for y/D ratios of 0.5 and higher. For smaller y/D ratios (0.063–0.25), DO levels stabilize after 1 and 3 km at just below 4 g O_2_/m^3^ and 1.5 g O_2_/m^3^, respectively. McCall et al. (2016b) report biofilm area to sewer volume ratios of 33 m^2^/m^3^ for real gravity sewers. A field study conducted by Gao et al. (2018) reports an AV ratio of 26 m^2^/m^3^ for a real pressurized sewer in Australia. However, most bioreactor studies use much larger AV ratios of 50–72 m^2^/m^3^. Also, spiking of compounds to higher concentrations as found in real wastewater could lead to artificially high removal rates as solvents like ethanol could serve as substrate for microbes and thus artificially enhance their activity (Hajj-Mohamad et al. 2017). On the other hand, wastewater temperatures in reality can exceed bioreactor temperatures with more than 10 °C, especially in subtropical regions and during summer months, which could translate into seasonally higher biotransformation rates. Results from bioreactor tests can provide a good indication of the maximum magnitude of in-sewer transformation that could occur, but differences between laboratory settings and the specific local circumstances need to be considered when extrapolating to realistic sewer conditions.

### Pilot sewers

Pilot sewers replicate real sewer systems at a larger scale than bioreactors, yet still controllable, using mainly PVC pipes at realistic slopes that convey real wastewater over multiple meters (Table 2). When recirculation tanks are used, travel distances can even increase to a few hundred meters or even kilometers, thus resulting in realistic residence times. Only four studies were identified using pilot sewers (Gao et al. 2019; Li et al. 2019a, b; Ren et al. 2021; Shi et al. 2018) which seems plausible given the financial investments and the space required to build such an installation.

**Table 2:** Overview of pilot studies assessing in-sewer stability of CECs

Reference	Compounds studied	Location	Wastewater used	Length sewer	Pipe diameter	Pipe material	Sewer type	AV ratio	T ww	pH	HRT	Sampled matrix	Spiked?	Triplicate?	Duration of experiment	Remarks
				[km]	[mm]			[m-1]	[°C]		[h]					
Gao et al. (2019)	Pharmaceuticals	Australia	Real, domestic	0.3	225	PVC	GS	27	21–24	7	4	Water	6 of 14 compounds	Yes	8 h	Both RM and GS conditioned for a year, with real influent ww resulting in mature biofilms
		Australia	Real, domestic	0.3	100	PVC	RM	40	21–24	7	8	Water	7 out of 14 compounds	Yes		
Li et al. (2019a, b)	Illicit drugs	Australia	Real, domestic	Same settings as Gao et al. (2019)					23	RM:7 GS:7.25	8	Water	yes, except for 2 (high background concentrations)	Yes	8 h	Same settings as Gao et al. (2019); rhodamine used as tracer; flow velocity in GS rather low (0.38 m/s)
Ren et al. (2021)	Consumer products	China	Synthetic	1.2	40	PVC	GS	n.a	n.a	n.a	n.a	Water + sediment	Yes, at 40–50 µg/L	no	90 days	Crucial parameters/settings not mentioned
Shi et al. (2018)*	Nutrients	China	Real, domestic; real sediment	0.032	200	n.a	GS	n.a	25**	n.a	n.a	Water + sediment	no	no	61 days	Assesses physical and biological pollutant transportation pathways (including equations)

*RM*, rising main (pressurized sewers), *GS*, gravity sewer; *AV ratio*, biofilm area to sewer volume ratio [m^2^/m^3^]; *AS*, activated sludge; *T ww*, temperature in wastewater; *ww*, wastewater; *HRT*, hydraulic retention time; *n.a.*, data not available in main article/supplementary information

*Additional publication on microbial community in same pilot

**unclear if water or air temperature

Even though data is currently limited, pilot sewer tests are more complex regarding the interplay of single fate processes as discussed in Sect. 3 than bioreactors. While experimental settings such as hydraulic retention time, pH and temperature can still be controlled to some degree, differentiation between single biochemical processes like sorption, biotransformation or back-transformation is increasingly difficult. For example, removing biofilms attached to the walls of several hundreds of meters of piping to study only abiotic processes could be challenging. The two Australian pilot-scale studies (Gao et al. 2019; Li et al. 2019a) replicated both gravity and pressurized sewer conditions and compared their results to previously conducted bioreactor tests for 6 compounds. Gao et al. (2019) observed the compounds being more stable at pilot scale than in bioreactors, suggesting that lab-scale tests indeed overestimate degradation as hypothesized in Sect. 4.3. They further observed that in contrast to most bioreactor tests, transformation kinetics for the studied compounds could not be fitted unambiguously to any kinetic model.

### Field studies

Our literature review identified only four studies that assessed stability of CECs in real sewers (Table 3). Three studies were performed in pressurized sewers while only one study was conducted in a gravity sewer (McCall et al. 2017). Two studies were conducted in the same sewer stretch (Gao et al. 2018; Li et al. 2018) while the study by Jelic et al. (2015) was conducted in a much longer (7 km) pressurized sewer resulting in a longer residence time. McCall et al. (2017) conducted a unique experiment comparing the impact of very mature biofilm (5 years old) in real gravity sewers on the transformation rates of several illicit drugs and their metabolites.

In the study by Jelic et al. (2015), no significant compound loss was observed between both sampling points for most of the 43 studied pharmaceuticals. Also Gao et al. (2018) observed lower degradation for compounds related to nicotine and alcohol consumption in a real sewer as compared to laboratory settings. For example, for ethyl sulfate a bioreactor half-live of 1.3 h was observed whereas the compound appeared to be stable under field settings. The authors attributed this to lower AV ratios in real sewers (about a third of bioreactor settings). Therefore, not just bioreactors imitating gravity sewers might overestimate dissipation due to higher O_2_ levels but also bioreactors imitating pressurized sewers might overestimate dissipation due to higher AV ratios.

**Table 3:** Overview of field studies conducted in real sewers to assess stability/degradation of CECs

Reference	Compounds studied	Location	Length sewer	pipe diameter	Pipe material	Sewer type	AV ratio	T ww	pH	HRT	Sampling method	Sampled matrix	Spiked?	Triplicate?	Remarks
			[km]	[mm]			[m-1]	[°C]		[h]					
Gao et al. (2018)	Alcohol, tobacco biomarkers	Australia	1.08	150	n.a	RM	26.7	n.a	n.a	1.5–6	Grab sample	Water	Only with tracers, not for study compounds	Yes	Rhodamine used as hydraulic tracer, acesulfame as benchmark tracer (O’Brien et al 2017). Li et al. (2019a): “obtained data points insufficient for kinetics evaluation”
Jelic et al. (2015)	Pharmaceuticals	Spain	7.6	50	n.a	RM	n.a	22	7.2–7.6	21	24 h composite, 5x	Water	No	No	
McCall et al. (2017)	Illicit drugs; metabolites	Switzerland	5.1	n.a	Stonewear	GS	11	13.9–15.3	7.6–8.0	2.5	24 h composite	Water	No	Yes	2 experiments conducted: once with mature (5 year old) biofilm, and once just after municipal cleaning of the biofilm. Dissolved oxygen concentrations reported at all measuring locations. AV ratio is relatively low because wastewater discharge was controlled to be constant (30L/s) but much lower compared to normal circumstances (200–800 L/s under dry weather flow)
Li et al. (2018)	Illicit drugs; metabolites	Australia	1.08	150	n.a	RM	26.7	23	8.5–7	3.6–5.4	Grab sample	Water	Yes, 2 groups one contained metabolites and one parent compounds	Yes	Same sewer as Gao et al. (2018). Li et al. (2019a): "obtained data points insufficient for kinetics evaluation"; Rhodamine and acesulfame used as tracers for mixing and leakage

*RM*, rising main (pressurized sewer); *AV ratio*, biofilm area to sewer volume ratio [m^2^/m^3^]; *AS*, activated sludge; *T ww*, temperature in wastewater; *HRT*, hydraulic retention time; *n.a.*, data not available in main article/supplementary information

### Recap: empirical data on sewer fate of CECs

Currently, empirical research on biotransformation of CECs is conducted mainly in the field of WBE. Experimental set-ups range from laboratory-scale bioreactors via pilot sewers to real sewers. With increasing level of experimental complexity, disentangling the contribution of the individual fate processes and environmental parameters to the overall dissipation of CECs becomes more challenging. The resulting DT_50_s or degradation rates lump all fate processes together leading to difficulties in differentiating biotransformation from sorption, ion-trapping and back-transformation. DT_50_s strongly depend on local circumstances leading to high variability in empirical DT_50_s determined under different conditions and limiting extrapolation from the set of findings to the prediction of CEC fate in real sewers. Laboratory-scale bioreactors typically do not capture this variability and tend to favor degradation. As a consequence, results from bioreactor tests can be useful to determine relative biodegradability, like in a ready biodegradability test, but estimated half-lives and degradation rate constants are likely to represent maximum values. More research is necessary to study the link between microbial community and biotransformation, e.g., using metagenomic techniques. Studying a more diverse set of CECs in pilot sewers could shed more light on the role and relevance of in-sewer degradation for both WBE as well as emission estimation studies.

## Integrated fate modelling of CECs in sewers

Over the past decade, several sewer models have been developed focusing on various spatial and temporal scales, specific wastewater parameters and sewer-related processes. For example, Vollertsen et al. (2015) developed a model to predict the production of hydrogen sulfide (sewer gas) and wastewater pH in the sewers of San Francisco; Li et al. ([Bibr CR59][Bibr CR60]) modelled real-time sewer flow in Australia under different weather conditions; Vezzaro et al. (2014) developed a dynamic sewer model including fate of CECs. This latter model was recently expanded by Delli Compagni et al. (2020) and considers in its latest version also back-transformation due to deconjugation and sequestration of CECs in feces. Other researchers recently reviewed in detail general fate models for various chemicals and their application domains (e.g. Su et al. 2019), the evolution of fate and exposure models (e.g. Bonnell et al. 2018; Di Guardo et al. 2018), as well as available sewer models to estimate wastewater parameters (Jia et al. 2021). To avoid duplication and in view of the aims of our study, we focus here on four models that have been specifically developed to model the fate of CECs in sewers. In this section, we compare these fit-for-purpose models to illustrate current approaches towards modelling fate of CECs in sewers. Hereto, we briefly describe common features and limitations, discuss alternative approaches, summarize the current status quo towards in-sewer modelling of CECs, and highlight future research needs.

### Fit-for-purpose modelling approaches

**Fig. 3:**
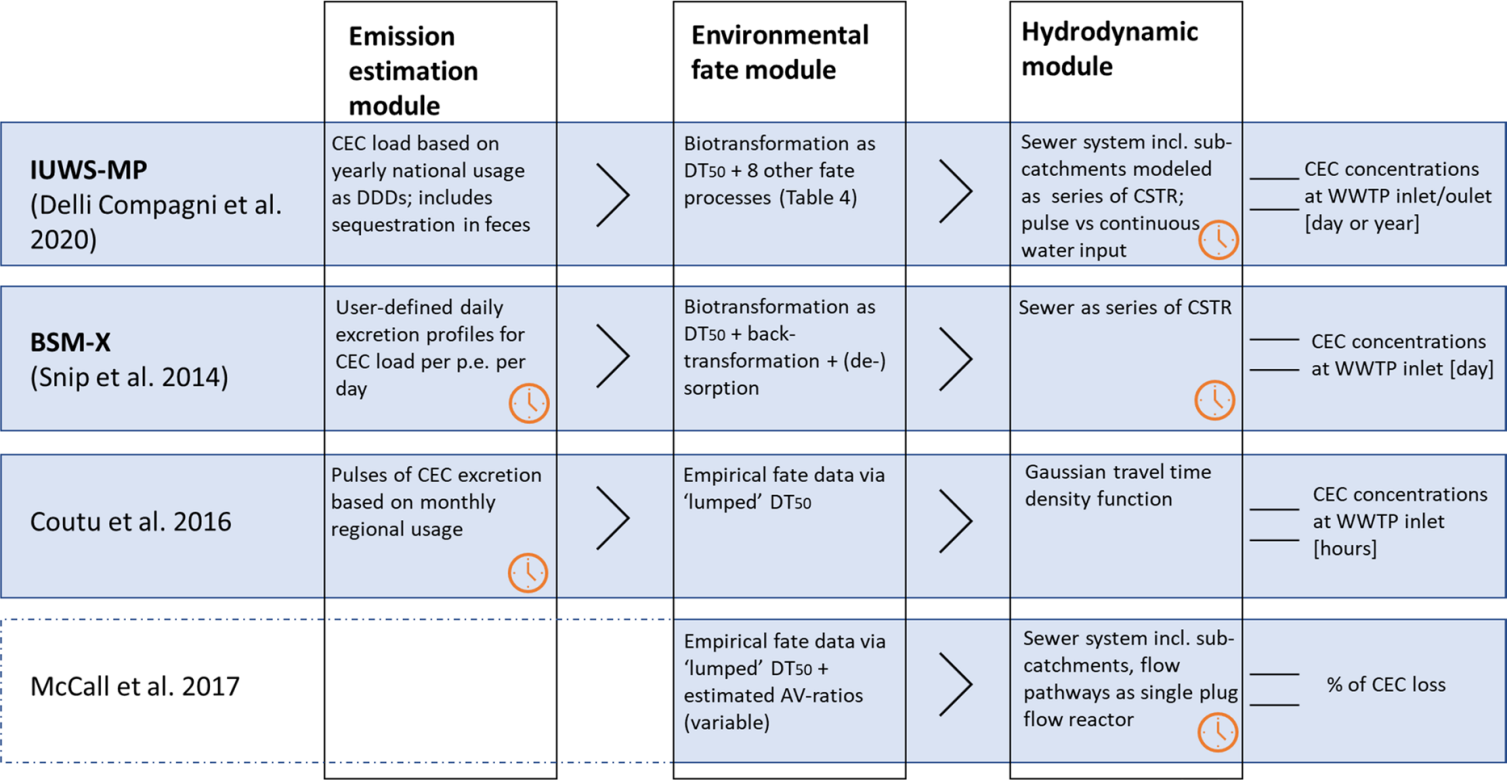
Schematic overview of model conceptualization. DDDs = daily defined doses; CSTR = continuously stirred tank reactor. Orange clock symbol indicates where the dynamic element of each model is implemented

In this section, we compare the selected models and discuss common features as well as limitations. To the best of our knowledge, the selected models (Fig. 3, Table 4) are currently the only ones available to model in-sewer fate of organic CECs as defined for this research. Three models were chosen from the review by Jia et al. (2021) and one more recent model was added upon reviewing recent literature (search terms in SI1).

**Table 4:** Overview of selected models and methods to estimate important parameters when modelling the fate of CECs in sewers

Model + Reference	Description/objective	Elements modelled	Validated?	Model performance
IUWS_MP Delli Compagni et al. (2020)	Dynamic model for fate of CECs in sewer systems implemented in WEST® software (DHI, Hørsholm, Denmark) based on ‘Activated Sludge Model (ASMd2)’	Hydrodynamic model to identify sub-catchments within sewer system for ‘pulse input’ or ‘continuous input’ under dry weather flow, sewer represented as series of continuously stirred bioreactors (Delli Compagni et al. 2019); Consumption-excretion model based on national consumption data including sequestration in feces; Fate module including under aerobic conditions: Biotransformation, (de-)sorption, ionization, hydrolysis, photolysis, volatilization, back-transformation, sequestration as first-order decay	Validated in two case studies (Italy + Denmark) in two real sewer systems using 24 h composite samples at sewer outlet (and WWTP outlet in one case study) under dry weather conditions for five pharmaceuticals (paracetamol, diclofenac, ibuprofen, furosemide and carbamazepine); Model calibrated based on TSS, NH4 in WWTP influent and sludge production, TSS and SRT in WWTP	Given results from case studies, model tends to overestimate WWTP influent as compared to measurements. Inclusion of sequestration improved overall model performance, while back-transformation and sequestration ‘led to the closest match between predictions and measurements in all parts of the systems’
BSM-X Snip et al. (2014)	Dynamic model based on ‘benchmark simulation model (BSM)’ in combination with the ‘Activated Sludge Model for Xenobiotic trace chemicals (ASM-X)’ to describe fate of pharmaceuticals in sewers and WWTPs on a daily basis	Hydrodynamic model represents sewer as series of continuously stirred bioreactors; Fate module including: (de-)sorption, back-transformation (aerobic, anoxic), biotransformation (aerobic, anoxic)	BSM and ASM-X have been validated separately, but BSM-X was not validated using measuring data (or at least not as part of Snip et al. 2014) Model applied to sulfamethoxazole, ciprofloxacin, tetracycline, diclofenac, and carbamazepine representing different administration pattern	In comparison to literature and other models, BSM-X seems to provide realistic simulations. Limitations are discussed in detail ‘Even though one might have the impression that the results are quite specific, the presented set of tools is quite generic and can be easily customized to represent different systems, on condition that sufficient data are available.’
McCall et al. (2017)	Stochastic model to quantify CEC loss based on estimated sewer residence time, AV ratio and in-sewer degradation of drug biomarkers. Implemented in EPA-SWMM	Sewer system implemented in EPA-SWMM; wastewater flow pathways modelled as single plug-flow reactor; sewer residence time was estimated a priori; AV ratios as a function flow; CEC degradation based on DT_50_s in combination with estimated varying AV ratios	Hydrodynamic module was validated using continuous flow data during dry-weather discharge and sewer catchment information of Dresden (Germany). Results from fate module were compared to measuring data for 14 compounds in Swiss sewer stretch with and without biofilm	Hydrological model seems to match closely with measured discharge during dry-weather Model was effective in estimating overall effect of biofilm (AV ratio) on in-sewer degradation, but validation of individual DT_50_s was limited
Integrated stochastic model for pharmaceuticals in sewage Coutu et al. (2016)	Stochastic model to ‘simulate [hourly] pharmaceutical concentrations at the entrance to a [W]WTP’	Excretion model to ‘simulate pulses of antibiotic mass into sewer network’; focusing on parent compound; Transport-degradation model lumping various fate processes into first-order decay; Hydraulic model as described in Coutu et al. (2012); “toilet flush frequency density function”	Model applied to sewer catchment in Lausanne (CH), using monthly sales data of antibiotic ciprofloxacin Model calibrated using four 24 h measuring campaigns (in Dec 2010; May, Sep and Nov 2011). Per campaign, 15 min grab samples were collected at WWTP influent and pooled per hour. One campaign conducted under rainy conditions, while others under dry weather	Under dry-weather conditions, model performed reasonable according to mass balance compared to empirical data at WWTP influent Did not perform well under rainy conditions

*WWTP*, wastewater treatment plant; *TSS*, total suspended solids; *SRT*, sludge retention time

Conceptually, all four models are composed of modules including at least a hydrodynamic module to generate the flow of wastewater in the sewer system and a fate module to assess effect of in-sewer dissipation (Fig. 3). Three out of four models have an additional emission module to estimate the loads of pharmaceuticals to wastewater. In the BSM-X model by Snip et al. (2014), for example, the pharmaceutical emissions are calculated as load per population equivalent and subsequently scaled according to the size of the contributing population. Similarly, the emission module in the IUWS-MP model uses yearly national usage data of pharmaceuticals expressed as daily defined doses (DDDs) to calculate emission loads to the sewer system via dosing schemes and excretion fractions. Coutu et al. (2016) follow the same principle to estimate the pharmaceutical input from monthly usage data into the sewer system but describe the human metabolism of pharmaceuticals using a set of differential equations, enabling the simulation of time-dependent excretion. The output of the emission estimation module (= excreted load to wastewater) serves as input for the respective fate modules.

Secondly, the fate modules of all selected models resemble each other. The models by McCall et al. (2017) and Coutu et al. (2016) both focus on in-sewer dissipation in a ‘lumped’ manner without discriminating between single fate processes such as sorption, biotransformation or deconjugation, using empirical DT_50_s as main input. The IUWS_MP and BSM-X models simulate biotransformation via DT_50_s but simulate most other fate processes mechanistically. These models describe in-sewer fate as a function of compound properties and wastewater characteristics: (de-)sorption of CECs is modelled via K_d_ values and total suspended solids (TSS) content of wastewater; or in the case of the IUWS_MP model ionization is modelled via pKa values and the pH of wastewater. Only the model by McCall et al. (2017) takes a more mechanistic approach towards modelling biotransformation by considering the effect of biofilm area in different pipe diameters via estimated AV ratios. The effect of back-transformation due to deconjugation on pharmaceutical loads is considered in the IUWS_MP model as well as in the BSM-X model. Since underlying mechanisms are not yet deterministically described, both models assume full deconjugation of metabolites into their respective parent compound. However, Delli Compagni et al. (2020) indicated that this assumption might overestimate pharmaceutical loads in WWTP influent. Furthermore, all models assume biotransformation to be the major process in affecting the load of CECs in sewer systems. However, since the underlying mechanisms of biotransformation are still poorly understood, all models rely on lumped DT_50_s. From all selected models, the IUWS_MP is probably the most ambitious in modelling individual fate processes as it includes besides biotransformation also ionization, sorption, back-transformation due to deconjugation, sequestration, hydrolysis, photolysis and volatilization (Table 4).

Regarding temporal resolution, all four models operate in a dynamic manner modelling the quality of wastewater over time in the order of minutes to hours. The dynamic character of the models is mainly dictated by the conceptualization of the underlying hydrological module and leading to conceptually complex and computationally demanding models that are difficult to apply without extensive background in hydrodynamics or dynamic modelling. Furthermore, hydrodynamic models require appropriate input data such as time series of rainfall, sewer discharge or drinking water consumption. The models by Coutu et al. (2016) and Snip et al. (2014) introduce a time-dependency in their emission estimation models as dosing and excretion pattern are considered per hour. Except for the model of McCall et al. (2017) that quantifies CEC loss, the sewer flow estimated via the hydrodynamic modules represents in all other models a time-dynamic dilution factor that is used to derive water concentrations based on CEC loads as modelled via stand-alone excretion and fate modules.

One main limitation is that validation of all discussed fit-for-purpose models is tricky for several reasons. Since all models are composed of different modules, preferably each of the modules would need their own validation step. To validate the predicted fate of CECs, either the mass balances of the modelled CECs (validation on consumption-excretion-fate model train) or predicted water concentrations at WWTP inlet (validation then also includes the hydrological model) can be compared to measured data. In both cases, if water samples are used for validation, only the dissolved fraction of modelled CECs can be validated as water samples typically miss sorbed fraction. The IUWS_MP and the model by Coutu et al. (2016) have been validated using measured CEC concentrations at WWTP inlets of the respective case study locations. According to the respective authors, both models performed reasonably well under dry weather flow. Estimated water concentrations for carbamazepine by the IUWS_MP matched measured concentrations, but for paracetamol the IUWS_MP overestimated water concentrations up to 60%. Only Coutu et al. (2016) attempted to also model wet weather flow but attributed the resulting mismatch between modelled water concentrations or loads of ciprofloxacin and measured data to the effect of desorption of sorbed ciprofloxacin as also observed in other studies (e.g. Bagnis et al. 2018). While their model did capture the dynamics in wastewater flow, it failed to describe the effect of desorption with its fate module lumping various fate processes into a single dissipation rate. Since the two mechanistic models (IUWS_MP and BSM-X) have not been applied to wet weather circumstances, it remains unclear if those models would have performed better under wet weather discharge given that both models include desorption. This highlights the need to validate models to various circumstances to assess their applicability range. None of the here described models takes sewer leakages or CSOs into account.

Furthermore, the presented models have only been applied to selected CECs. Altogether, the four models were applied to 11 pharmaceuticals and 9 compounds related to illicit drug consumption (Table 4). Diclofenac and carbamazepine have been modelled by all models except for the one by Coutu et al. (2016); ciprofloxacin has been modelled by Coutu et al. (2016) and Snip et al. (2014). Due to the different conceptualization and the specific case studies, it is difficult to compare the model performances also because model performance is not always quantified by the respective authors. Furthermore, the presented models have not yet been applied to a broader range of CECs. Therefore, it remains unclear how well these models perform for compounds representing a wide chemical spectrum. This, however, would be crucial to assess the validity and application options for such models especially regarding ‘special groups’ of CECs such as ionizing compounds.

### Alternative modelling approaches

If we let go of hydrodynamics and focus on fate processes in relation to sewer residence time, a much simpler and more flexible approximation is possible. Besides compound characteristics required to estimate their fate (K_d_, log K_ow_, pK_a_, DT_50_s), sewer characteristics need to be considered. With the current approach to model biotransformation as DT_50_s, sewer residence time is by far the most important parameter. To estimate catchment specific residence times, the stochastic approach by McCall et al. (2017) and Delli Compagni et al. (2019) or a GIS-based approach as described in Kapo et al. (2017) could be applied. Especially the approach taken by Kapo et al. (2017) to estimate sewer residence time for gravity sewers based on the road network of a city is relatively straight-forward and easy to implement in ArcGIS. For pressurized systems, such estimation methods do not yet exist to the best of our knowledge and residence times would need to be determined based on system characteristics and expert estimates in combination with water consumption data.

A high temporal resolution of the hydrodynamic module is not always needed. If the research aim is to estimate the peak exposure of local surface water due to combined sewer overflow emissions, wastewater flow needs to be modeled at a high temporal scale. In contrast, hydrodynamics become less important if the model is used, e.g., to study the long-term effect of different emission reduction strategies on the CEC load entering a WWTP. When studying such static phenomena, a multimedia fate approach could be enough to describe in-sewer fate of CECs. Since the 1990s, a number of multimedia fate models have been developed to assess the behavior of various chemicals in the environment for different spatial scales based on fundamental physical/chemical laws. Most multimedia fate models simplify and generalize complex interactions of chemicals and the environment based on the fugacity approach as described by (Mackay 2001). A prominent example of such model is SimpleBox (Schoorl et al. 2015), which assesses the fate of chemicals in various environmental compartments such as air, water, soil and sediment on different spatial scales at steady state. SimpleBox is used for regulation of chemicals in the EU and served as theoretical concept for SimpleTreat (Struijs 2014) which predicts the fate of chemicals in WWTPs. Although SimpleTreat has not yet been applied to sewer systems directly, the conceptualization of the model could be used to develop a simple sewer fate model in combination with estimated sewer residence times. Mean sewer residence time could be accounted for by adapting the hydraulic retention time (HRT) within SimpleTreat. If more specific information on the sewer system is available, a more refined approach could be implemented to model sub-catchment and related residence times as a series of activated sludge basins similar to the approach described in Delli Compagni et al. (2019). However, the performance of SimpleTreat is suboptimal as shown in a number of studies (e.g., Douziech et al. 2018; Lautz et al. 2017). As indicated by Douziech et al. (2018), the model performed better if the order of magnitude of the influent concentrations was known. However, it remains unclear if the poor performance of SimpleTreat is a result of modelling or measuring uncertainties as neither the model nor the measured WWTP removal take the effect of deconjugation into account. This stresses the need for more research into sewer and WWTP fate processes, particularly regarding back-transformation.

### Recap: integrated fate modelling of CECs in sewers

The ideas and aims behind each of the models discussed are valid and continue to be of high scientific and societal interest (e.g., also in the light of wastewater surveillance regarding Covid-19). The dynamic character of the here discussed fit-for-purpose models relates to the underlying hydrological modules leading to limited applicability because hydrodynamic modules are very data-driven, computationally demanding and require advanced modelling skills. Besides, the most important in-sewer fate processes are not yet sufficiently well understood to enable these models to live up to their expected applications. To improve fate modelling, especially biotransformation and back-transformation need to be studied in more detail as using empirical or estimated DT_50_s to approximate biotransformation represents the main limitation in all these modelling approaches given DT_50_s are extremely variable and context-dependent (see Sect. 4).

Given that the fate modules are conceptualized in a much simpler way, and that sewer flow basically represents a dynamic dilution factor, it is questionable whether model performance will improve by focusing on reducing uncertainties around sewer hydrology. Instead, a better mechanistic understanding of the fate processes could substantially improve model performance and applicability. Depending on the research context, much simpler models will do the job probably equally good as the complex, dynamic models presented here. For example, the fate module of the complex IUWS_MP model is almost identical to the steady-state model SimpleTreat and thus relying on the same input data. Once mechanistic understanding of biotransformation in sewers and wastewater is improved and validated, we should first validate the fate modules using mass balances and empirical sewer measurements.

## Conclusions and recommendations

### Main conclusions from this literature review

The aim of this literature review was to compile detailed information on the fate of CECs in sewer systems, to eventually enable quantification of environmental loads given urban chemical use statistics or enable quantification of urban use given loads received at WWTPs. When estimating wastewater-related CEC emissions from urban areas to the environment, the sewer system can play an important role. When comparing the load of CECs discharged into the sewer system with the load reaching the WWTP inlet, the passage through the sewer system can lead to three possible outcomes:

- discharged CEC loads equal CEC loads at the WWTP inlet;
- a decrease in CEC loads at the WWTP inlet;
- an increase in CEC loads at the WWTP inlet.

Which of the three outcomes is most likely for a particular CEC depends on compound properties and characteristics of the sewer system. Local circumstances regarding the sewer infrastructure, climatic pattern and factors causing variability in wastewater flow need to be considered when estimating wastewater-related CEC emissions from urban areas to the environment.

Regarding in-sewer fate processes, all biochemical processes discussed above are relevant for the fate of CECs in sewers. The mechanistics of sorption of CECs as a function of lipophilicity and ionization state is relatively well understood, but the applicability of traditional approaches to model sorption in wastewater needs to be revisited. While K_ow_ values adequately reflect sorption as a result of lipophilicity to organic matter, the parameter appears to be a poor proxy to estimate sorption potential of a compound in wastewater. This is mainly due to the composition of wastewater, which contains high protein contents, suspended particles, sewer sediments and biomass and therefore differs substantially from octanol. The composition of wastewater requires more empirical research especially regarding the contents of organic carbon and of other components such as proteins and charged particles that could affect sorption of CECs. Biotransformation, ion-trapping and deconjugation are poorly understood. While links with general parameters such as temperature and biomass abundance are known, more specific knowledge is required to disentangle the contribution of each single process to the overall in-sewer fate of CECs. More specifically, the mechanistic links between biotransformation and the composition of microbial communities in wastewater, biofilms and sewer sediment need to be studied in more detail. Attention needs to be paid to seasonal and regional variability of these conditions between sewer systems as well as within single sewer catchments. The current approach in modelling biotransformation as lumped dissipation rate or DT_50_ is extremely limited as high variability in empirically derived DT_50_s for individual compounds limits the applicability of DT_50_s between studies. For these reasons, the accuracy in modelling in-sewer fate of CECs is still limited even though a number of integrated sewer models have been developed over the past years.

### Rules of thumb for addressing sewer fate processes

Based on the currently available knowledge on fate processes affecting the load of CECs in sewer systems, we here present a set of pragmatic rules of thumb that can be used by water professionals to assess under which circumstances in-sewer fate needs to be considered. Generally, water managers can assume CEC input to the sewer system equals WWTP input (the above option ‘a’), unless one of the following criteria is met:

CEC loads can decrease towards the WWTP for

- Compounds having empirical DT_50_s ≤ sewer residence time as this indicates that in-sewer fate processes can affect the mass load of these compounds and should therefore be taken into account.
- Neutral compounds that have high K_d_ or K_oc_ values as these compounds will sorb. Sorption to sewer sediment and biofilm result in higher residence times of sorbed CECs and could lead to subsequent biotransformation. Sorption of CECs to suspended solids and biomass does not necessarily increase their sewer residence time, but could affect analytical results, for example, if water samples are being filtered before analysis.
- Bases with pKa values of 8.5–10 as these are mainly present as positively charged cations at typical wastewater pH and could therefore sorb electrostatically to biomass or organic carbon. Fate pathways for those compounds are thus comparable to neutral compounds with high K_d_ or K_oc_ values (previous point).
- Amine-containing compounds with pKa values between 7–10 and log K_OW_ of 0.4–4.2 as these compounds might become ‘trapped’ within sewer organisms.
- Sewer catchments in regions where during extended periods wastewater temperatures of 15–35 °C are observed as biotransformation would proceed faster than indicated by most experiments to derive DT_50_s.
- Sewer catchments with damaged sewer pipes in areas with low groundwater tables or close to drought-stressed riverbanks, as wastewater could leak from the sewer system to the surroundings.

CEC loads can increase towards the WWTP for

- Pharmaceuticals (and other substances) that are excreted as conjugated metabolites especially glucuronides, since these metabolites can back-transform within few hours to their respective parent compounds.
- Combined sewer systems in regions with seldom but heavy rainfall, as remobilization and desorption of CECs in sewer sediments can become important. Periods of prolonged drought favor the development of sewer sediment due to low discharge and more concentrated wastewater. Once sewer discharge increases, e.g., after a heavy rain event, the deposited sewer sediment can resuspend affecting previous sorption equilibria due to dilution and ‘fresh’ particulate matter that had accumulated on streets and surfaces.

In these situations, the water manager should make an effort to quantify the impact of in-sewer fate processes when estimating CEC emissions. If this is not feasible, e.g. by modelling approaches described in this review, a worst-case assumption can be made in order to arrive at a conservative exposure estimate. In the case of back-transformation due to deconjugation for example, it could be assumed that all conjugated metabolites deconjugate and re-transform into their respective parent compounds.

### Recommendations for research

As highlighted throughout this review, a number of knowledge gaps exist around in-sewer fate processes, composition of wastewater, environmental conditions in sewer systems and the variability of each of these. Here we summarize our main suggestions for further research. We distinguish between research into the mechanisms driving in-sewer fate processes and research into the implications of in-sewer fate processes for environmental modelling.

An important mechanistic driver of in-sewer fate requiring additional research is the relationship between the composition of microbial communities in sewers and biotransformation rates. Meta-genomic profiling could help to assess temporal and spatial variability of the microbial community in sewers and their activity between sewer systems and within single sewer catchments. Similarly, the effect of ion-trapping on CEC loads in sewer systems and WWTPs requires more fundamental research. Another important mechanistic driver requiring further research is the enzymatic deconjugation of conjugated metabolites. Key questions include: What other microbes besides *E.coli* secrete enzymes capable of cleaving glucuronide additions of CEC molecules? In which sewer compartment do these enzymes occur and in what abundance? How do environmental parameters and wastewater composition affect enzymatic activity? What are the kinetics behind this process? Future studies should consider the sewer system and WWTP as one system consisting of two parts, as fate processes occurring in sewers affect WWTP inflow. Finally, the relation between sorption and the composition of wastewater, biofilms and sewer sediments needs to be studied further, particularly for charged/ionized compounds.

In relation to sewer modelling, traditional concept of bioavailability in relation to sorption and subsequent biotransformation of CECs requires critical re-evaluation. This concept is mainly based on soil-chemical interactions and assumes that sorption inhibits bioavailability and therefore biotransformation. Given that the composition of wastewater and other sewer compartments (previous point) substantially differs from the composition of soils, especially regarding the abundance and activity of biomass, it remains unclear to what extent the traditional approach is applicable to sewer environments. Similarly, bioavailability in relation to toxicity of CECs needs to be assessed for sewers and WWTPs. Another point of attention for sewer fate modelling, is the disentanglement of compound properties from environmental parameters and observed environmental fate. Creating a public database of DT_50_s and degradation rates of CECs for different environmental media end conditions could be a first step. Furthermore, results of OECD tests should become publicly available as these tests help to pin-point the effect of biotransformation in dissipation of compounds. Finally, more research is needed into the role of sewer infrastructure in CEC emissions, especially assessing the role of combined sewer overflows and sewer leakages. Similarly, continuous emissions of CECs from surface water run-off discharged via separated rainwater sewers should be evaluated in more detail.

The potential of wastewater-based epidemiology and the resulting ‘Big Brown Data’ became evident during the SARS-CoV-2 pandemic. Many countries adopted monitoring programs at WWTP inlets to estimate prevalence of Covid-19 among the population (Medema et al. 2020; Wade et al. 2022). Harvesting the insights obtained during the pandemic can help to strengthen wastewater surveillance programs not just for other infectious diseases but also for other potential threats to public health such as the spread of antimicrobial resistance. More research into the variability of parameters related to the sewer system could help to improve the accuracy of such ‘backward estimations’, i.e. estimating population exposure based on wastewater monitoring, but could also facilitate estimating human exposure to compounds for which no accurate usage or consumption data are available (e.g. industrial compounds). On the other hand, a better understanding of in-sewer fate processes would help to improve ‘forward estimations’ to assess environmental exposure and related risks based on consumption and usage data of CECs. Therefore, improving sewer (fate) models in combination with expanding wastewater monitoring programs to CECs would help to gain knowledge on trends occurring at both ends of the sewer system.

### Recommendations for wastewater management

Sewer systems and WWTPs are crucial infrastructure for society. Wastewater-related CEC emissions can play an important role in local surface water quality. To aid decision-making, this literature review aimed to compile relevant and comprehensible information for water managers on how to deal with in-sewer fate of CECs. In case of doubt, we recommend following the precautionary principle in making conservative (i.e., worst-case) assumptions when estimating environmental exposure to CECs.

Furthermore, investing in sewer-related research is recommended. Given that our current understanding of in-sewer fate processes is still limited, more research is needed to assess the interactions of CECs within the complex and variable ecosystem of sewer systems. A better mechanistic understanding of CEC fate in sewers could not only help to improve integrated modelling approaches but would also offer more efficient monitoring and prioritization of emission reduction efforts.

With increasing demands for clean water, there is a need to develop more innovative concepts to wastewater transport and treatment. We need to rethink sewer systems to improve their functionality from merely transporting to pre-treating wastewater. This might be particularly interesting in the light of asset management given that maintaining aged sewer systems requires significant public investments.

## Supplementary Information

Below is the link to the electronic supplementary material.

Supplementary file1 (DOCX 4002 KB)

Supplementary file2 (XLSX 34 KB)
