# Supplementary material for: The role of the sewer system in estimating urban emissions of chemicals of emerging concern
Source: Rev Environ Sci Biotechnol. 2022 Oct 23;21(4):957–91. doi: 10.1007/s11157-022-09638-9 (PMC9589831; doi:10.1007/s11157-022-09638-9)
Supplement: Supplementary file 1 — Supplementary file1 (DOCX 4002 KB) [file 11157_2022_9638_MOESM1_ESM.docx]

# Supplementary information

The role of the sewer system in estimating urban CEC emissions of chemicals of emerging concern: a review

Caterina Zillien^1*^, Leo Posthuma^1,2^, Erwin Roex^3^, Ad Ragas^1^

*^1^Radboud University, Department of Environmental Science, Nijmegen, the Netherlands*

*^2^ National Institute for Public Health and the Environment (RIVM), Centre for Sustainability, Environment and Health, Bilthoven, the Netherlands*

*^3^National Institute for Public Health and the Environment (RIVM), Centre for Zoonoses and Environmental Microbiology, Bilthoven, the Netherlands*

*Corresponding author: [caterina.zillien@ru.nl](mailto:caterina.zillien@ru.nl), ORCID ID: 0000-0002-3224-8866

20 pages

13 Figures

Contents

[Supplementary information to section 2 ‘Sewers and their characteristics’ 1](#_Toc114908021)

[Supplementary information to section 3.2 ‘Data analysis sewer half-lives’ 6](#_Toc114908022)

[Data collection 6](#_Toc114908023)

[Equations used to transform k values into half-lives for different kinetic models 6](#_Toc114908024)

[Data analysis performed using compiled dataset 7](#_Toc114908025)

[PCA and clustering 16](#_Toc114908026)

[PCA of DT50s and experimental settings 16](#_Toc114908027)

[PCA of aggregated DT50s and compound properties 18](#_Toc114908028)

[Search terms section 5 ‘Integrated fate modelling of CECs in sewers’ 19](#_Toc114908029)

[References 20](#_Toc114908030)

**List of Figures**

[Figure S 1 - Mode of transportation of 30 European sewer systems 1](#_Toc101975094)

[Figure S 2 - Geographical distribution of sewer survey conducted by Ort et al. 2014. 2](#_Toc101975095)

[Figure S 3 - Linear regression plot assessing maximum distance to WWTP and mean residence time 3](#_Toc101975096)

[Figure S 4 - Wastewater constituents measured at WWTP inlet of various European cities 4](#_Toc101975097)

[Figure S 5 - Observed temperature differences of WWTP influents 5](#_Toc101975098)

[Figure S 6 - Observed differences in pH of WWTP influents 5](#_Toc101975099)

[Figure S 7 - Reported DT50s according to type of study and prevailing redox conditions 8](#_Toc101975100)

[Figure S 8 - Compounds and their observed DT50s per study type and redox conditions 9](#_Toc101975101)

[Figure S 9 - Compounds with at least one half-life for 2 different study types per redox condition. 10](#_Toc101975102)

[Figure S 10 - PCA of individual DT50s and experimental settings. 16](#_Toc101975103)

[Figure S 11 - Correlation matrix of DT50s and experimental settings 17](#_Toc101975104)

[Figure S 12 - PCA on individual compounds and compound properties 18](#_Toc101975105)

[Figure S 13 - Correlation matrix of aggregated DT50s and compound properties 19](#_Toc101975106)

## Supplementary information to section 2 ‘Sewers and their characteristics’

The plots presented in this section are all based on the dataset compiled by Ort et al. (2014). Data analysis and visualization was done in R (version 3.6.1, R Core Team, 2019) using the following packages: tidyverse (Wickham, Averick, Bryan, & ..., 2019), ggplot2 (Wickham, 2016), dplyr (Wickham, François, Henry, & Müller, 2021), ggvis (Chang & Wickham, 2020), scales (Wickham & Seidel, 2020), Hmisc (Harrell, Dupont, & ..., 2021), RColorBrewer (Neuwirth, 2014), maps (Becker & Wilks, 2018b), mapdata (Becker & Wilks, 2018a), rworldmap (South, 2011) and mapproj (McIlroy, 2020).


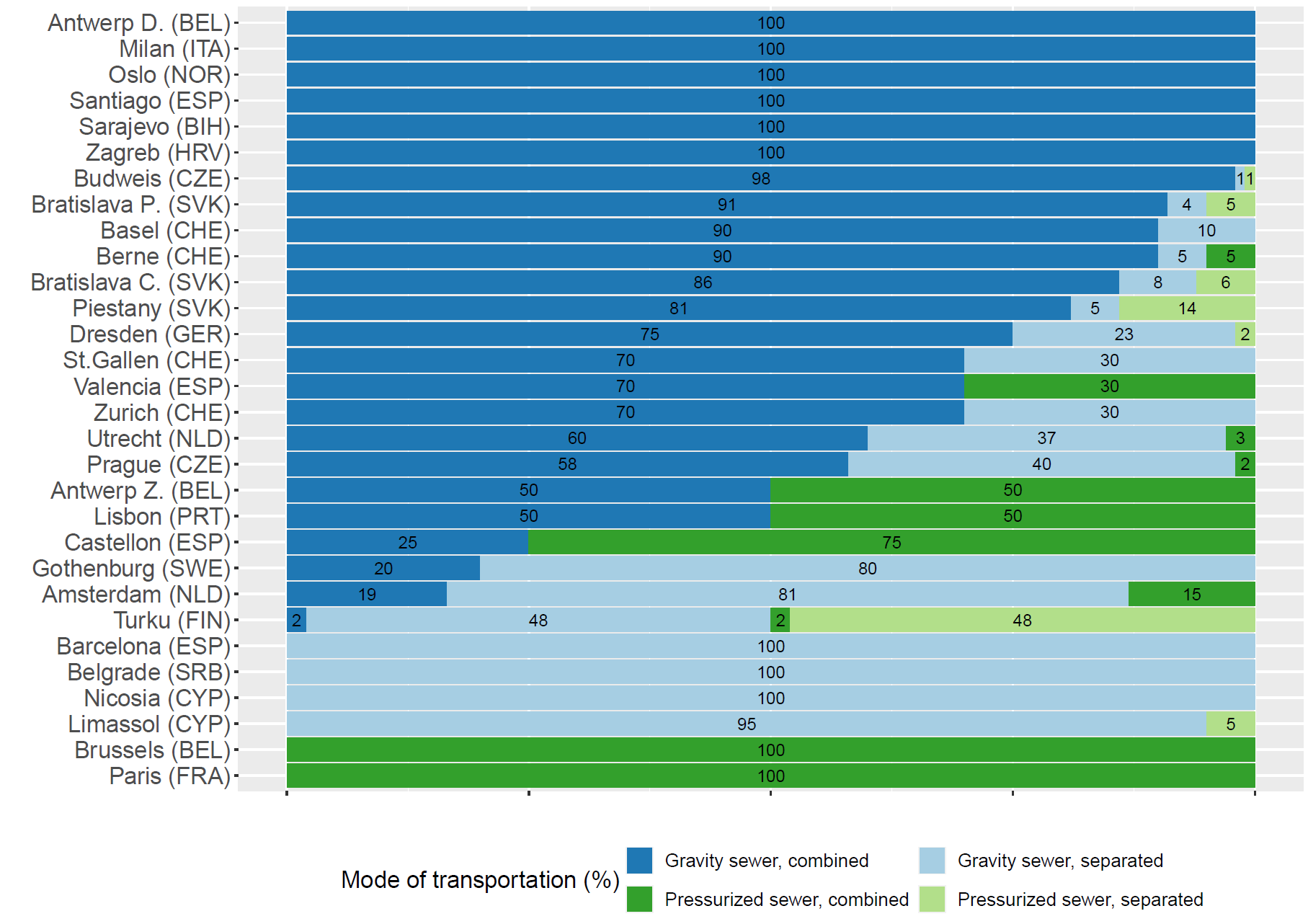


Figure S 1 - Mode of transportation of sewer systems connected to 30 European WWTPs. Data retrieved from Ort et al. 2014.


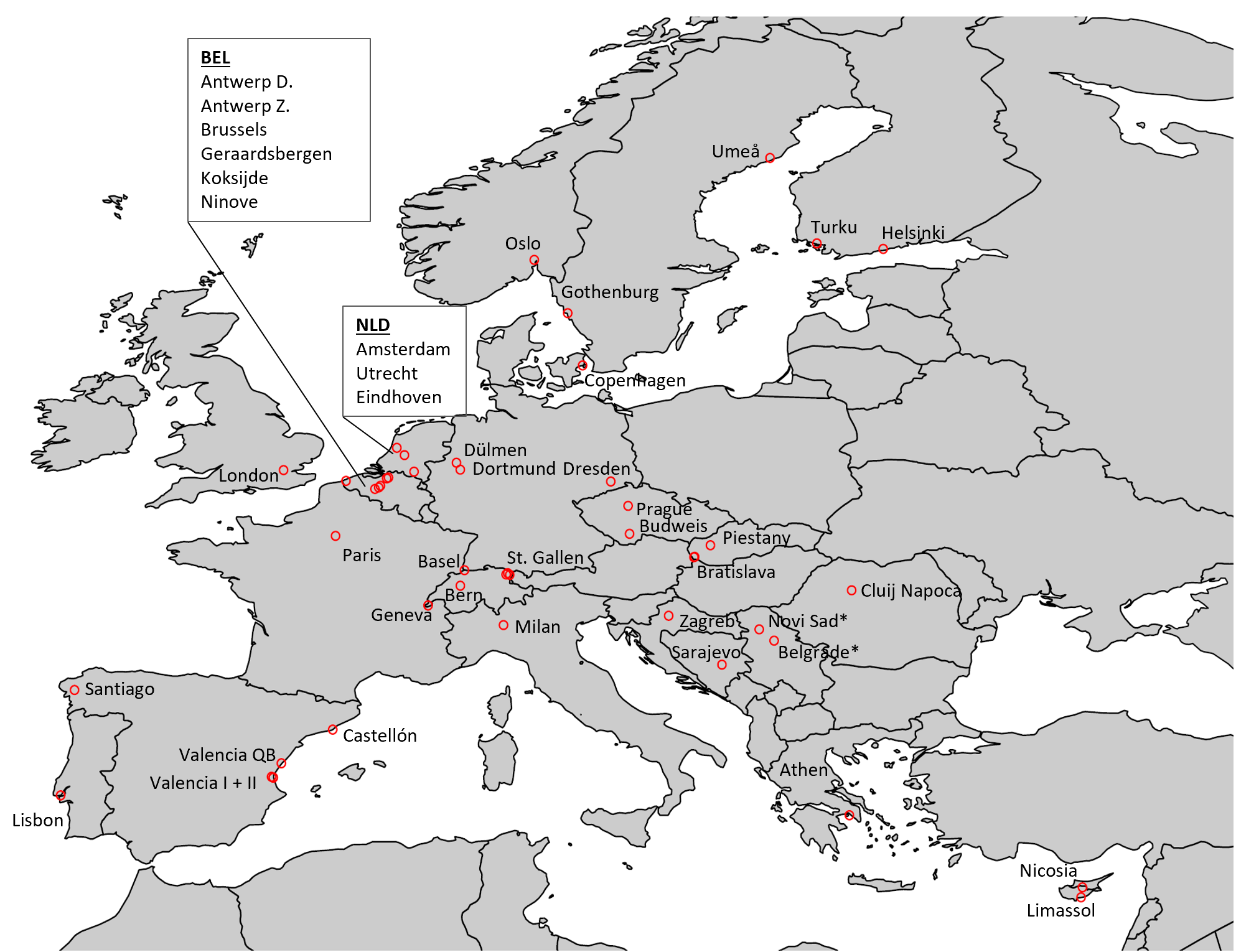


Figure S 2 - Geographical distribution of cities participating in sewer survey conducted by Ort et al. 2014.


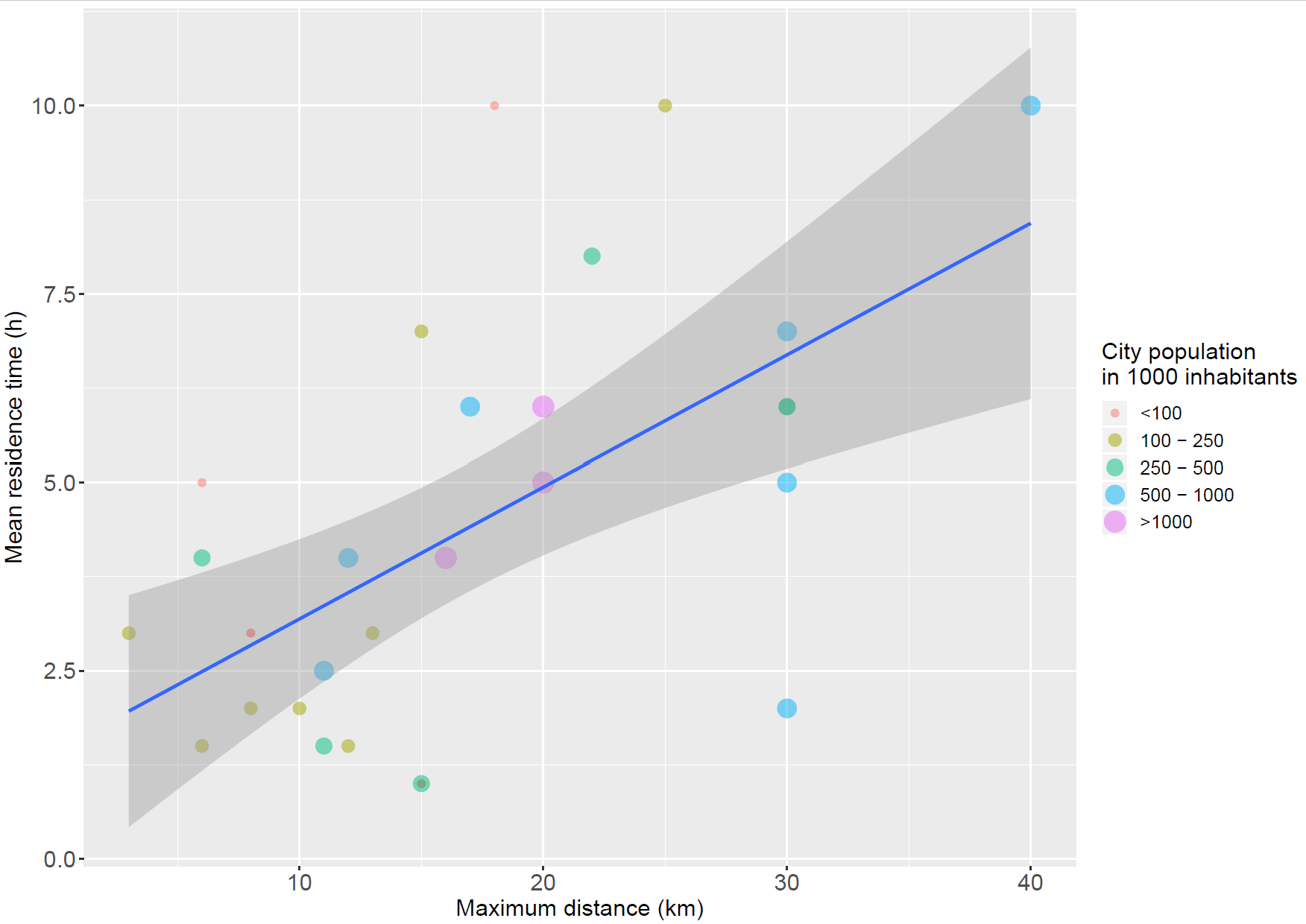


Figure S 3 - Linear regression plot assessing the relation between maximum distance (in km) within a sewer system to the closest WWTP and the mean residence time of this sewer system (in hours). Simple regression analysis showed a positive relation between residence time and maximum distance to WWTP (p-value = 0.000653, R-squared = 0.344). Data retrieved from Ort et al. 2014.


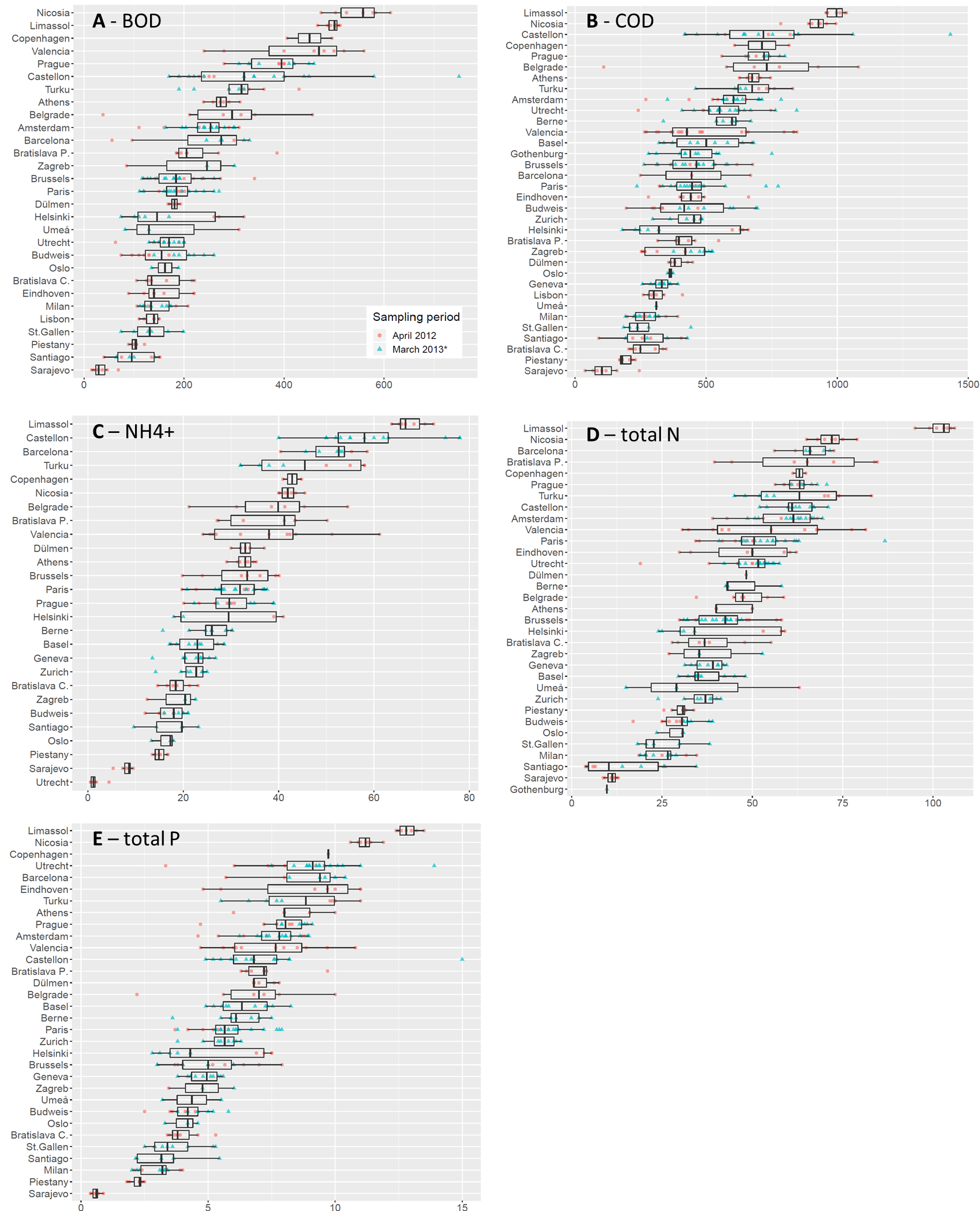


Figure S 4 – Wastewater constituents in mg/L measured at WWTP inlet of various European cities during two sampling campaigns in two consecutive years. *Sampling period varied in 2013 between March 6 (earliest) and March 27 (latest). Data retrieved from Ort et al. 2014.


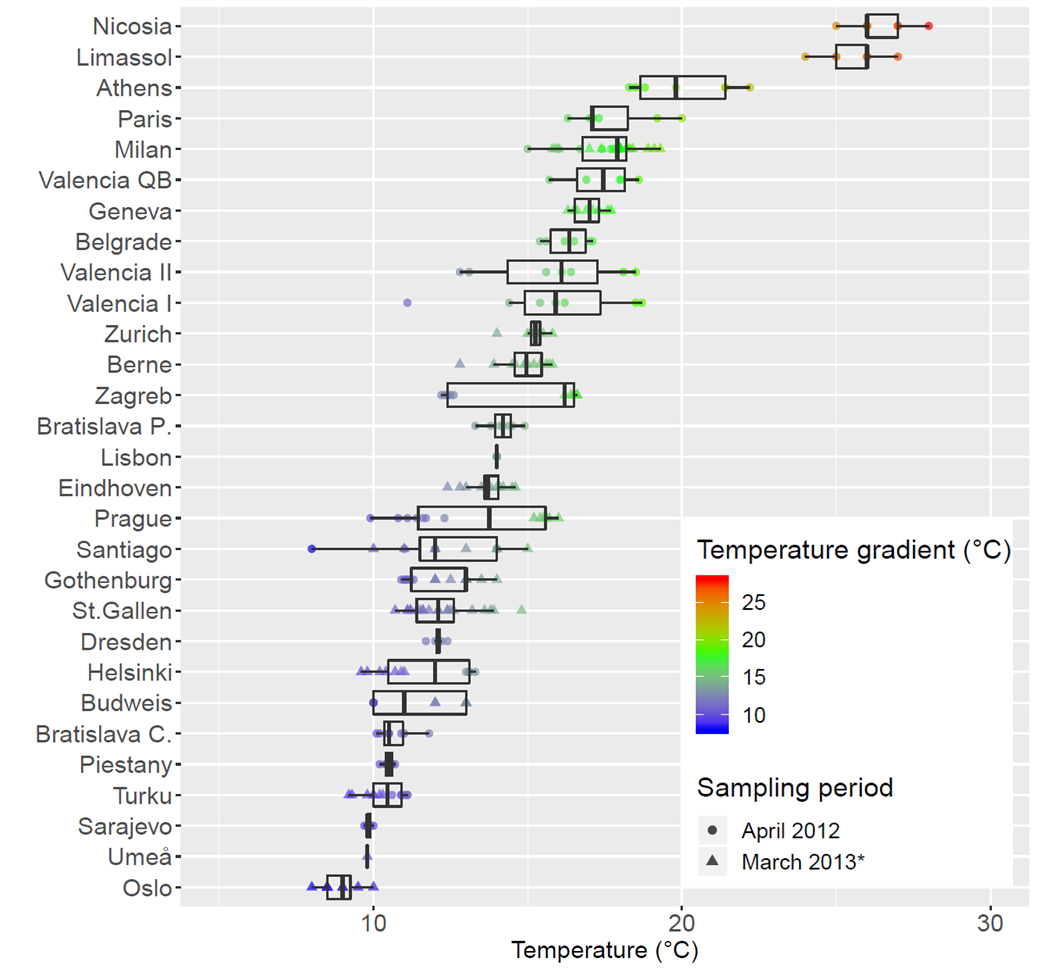


Figure S 5 - Observed temperature differences of WWTP influent during up to 15 consecutive days in spring 2012 and 2013, based on Ort et al. (2014). *Sampling period varied in 2013 between March 6 (earliest) and March 27 (latest).


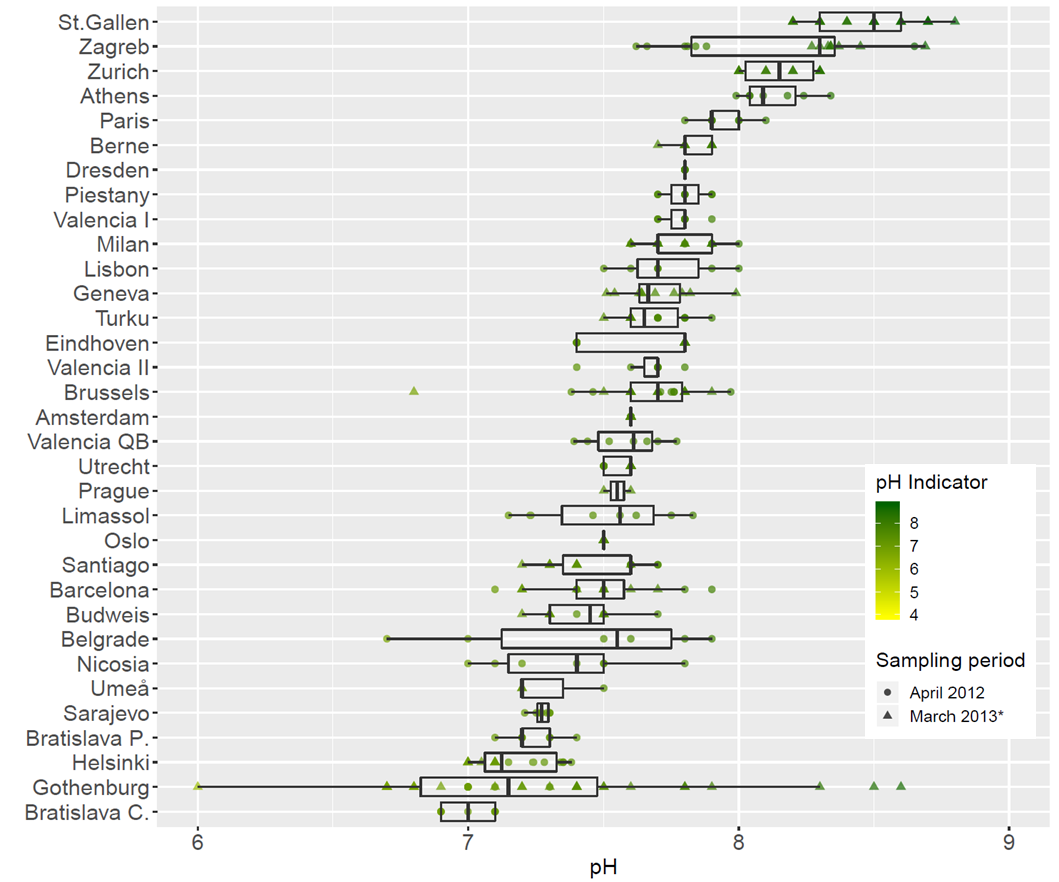


Figure S 6 - Observed differences in pH of WWTP influent during sampling periods in spring 2012 and 2013. Based on Ort et al. (2014). *Sampling period varied in 2013 between March 6 (earliest) and March 27 (latest).

## Supplementary information to section 3.2 ‘Data analysis sewer half-lives’

### Data collection

In order to compile most recent data on sewer stability of CECs, we conducted an extensive literature review in Web of Science ([www.webofscience.com](http://www.webofscience.com) ) using the following search terms between December 2020 and February 2021:

1. (in-sewer OR sewer OR sewage ) AND ((half-li* OR half liv* AND (degrad* )) AND (CEC* OR micropollut* OR pharmaceutic*): 74 results
2. ((lab OR pilot OR real) AND (sewer OR wastewater) AND (stability OR stab*) AND (pharmaceutic* OR biomarker* OR drug* OR CEC OR micropollut*)): 253 results
3. (((sewer*  OR wastewater  OR sew*)  AND (stability  OR stab*)  AND (pharmaceutic*  OR biomarker*  OR drug*  OR CEC  OR micropollut*)  NOT (remov*  OR treat*))): 262 results

When screening the abstracts, articles on field, pilot and lab scale focusing on organic CECs were considered useful. WBE studies and modelling studies were selected. Additionally, references of particularly useful articles were scanned to double-check for potentially missed studies.

**Argumentation on excluding stability studies from data collection**

*Excluded bioreactor studies*

Brown et al. (2020) used primary effluent (and other) of WWTP in Winnipeg (Canada) to assess stability of several pharmaceuticals throughout water treatment. One set of bioreactors was operated under aeration, the other set was not (only stirred). Measurement points were after 2h (to represent HRT of primary treatment) and 24h (to represent ‘lagoon’ circumstances). No data on dissolved oxygen (DO) provided, no R2 provided to judge the fit of first order model which was applied to derive t ½. Looking at the graphs displaying the stability (SI), neither first nor zero order regression seem to fit well. 🡪 Data points not included.

Similarly, study by Cormier et al. (2015) assessed stability in surface water and WWTP effluents 🡪 data not included.

Ramin et al. (2017) uses same data as reported in Ramin et al. (2016), so to avoid duplicates the 2017 study was not included.

*Excluded pilot studies*

Shi et al. (2018) investigated exchange of nutrients between sewer sediments and the water phase in a pilot setting. Since no CECs were assessed, this study was excluded from data collection.

Ren et al. (2021) assessed stability of CECs in a pilot setting, however information on crucial parameters such as water temperature, water pH or HRT were not reported. Furthermore, the study used synthetic wastewater and was therefore excluded.

### Equations used to transform k values into half-lives for different kinetic models

*Equation 1:* zero-order half-life $t\frac{1}{2}=\frac{C_{0}}{2k}$

*Equation 2:* first-order half-life $t\frac{1}{2}=\frac{ln(2)}{k}$

*Equation 3*: second-order half-life $t\frac{1}{2}=\frac{1}{k*C_{0}}$

**Study-specific methods to calculate half-lives from reported rate constants (k)**

Gallé et al. (2019) used “native activated sludges from the different treatment plants […] to determine elimitation raes for the MP under controlled conditions […] the reactors served to do respirometric tests for biomass characterization. DO was kept at 3 to 6 mg/L. Samples were taken over 7 hours. R2 and p values provided, results from duplicate tests (SI, Figure 14). 🡪 use k biol values as reported in SI Fig. 14 if R2 >0.5 and p <0.05. If both values fulfill criteria, then take average of both.

Data from (McCall, Palmitessa, Blumensaat, Morgenroth, & Ort, 2017; McCall et al., 2016) retrieved as reported in study from 2017. Data was apparently generated during the study in 2016 but reported in a more useful way (aggregated k values) in the 2017 study.

Ramin et al. (2016) assessed stability in 3 sets of batches: a) biotransformation using raw wastewater, b) sorption experiments with diluted primary sludge and c) abiotic experiments using mineral water. Consider here only results from a). Batches were aerated with oxygen (aerobic) or nitrogen (anaerobic) and continuously stirred. DO levels not reported. Different k values for biotransformation/abiotic processes. For biotransformation, they assume in WATS model second order decay. 🡪 sum of k bio and k abio divided by 24h to transform to /hour, then fit into 2nd order t ½. This approach is also followed by the authors (see caption to Fig. 2 in main article).

### Data analysis performed using compiled dataset

Data on half-lives of CECs in wastewater and sewers and experimental settings were collected manually from previously selected articles. Selected compound properties were collected from PubChem (log K_ow_, molecular weight) and scientific literature (log K_d_). Preference was given to experimental data over estimated values. The data collection and cleaning were performed in Excel. The resulting dataset contained 277 entries with half-lives for 96 unique compounds (SI1). 120 half-lives are shorter than 12 hours and can thus be considered relevant for urban sewer catchments. The vast majority of entries (100 of 120) were reported for bioreactor tests, while only few entries were reported for pilot and field studies (12 and 8 respectively).

1. **General description of dataset:**

Dataset contains 277 entries (= half-lives) for 96 unique compounds.

Far the most half-lives are from bioreactor studies:

type_study coumpound

*<fct>* *<int>*

1 bioreactor 246

2 pilot 14

3 real_sewer 17

Most of the bioreactor half-lives have been derived under aerobic conditions; half-lives for real sewers are only available for anaerobic conditions:

type_study redox_condition coumpound

*<fct>* *<fct>* *<int>*

1 bioreactor aerob 117

2 bioreactor anaerob 98

3 bioreactor control 31

4 pilot aerob 7

5 pilot anaerob 7

6 real_sewer anaerob 17

120 half-lives are <12h (=potentially relevant for sewer systems):

type_study redox_condition coumpound

*<fct>* *<fct>* *<int>*

1 bioreactor aerob 43

2 bioreactor anaerob 49

3 bioreactor control 8

4 pilot aerob 5

5 pilot anaerob 7

6 real_sewer anaerob 8


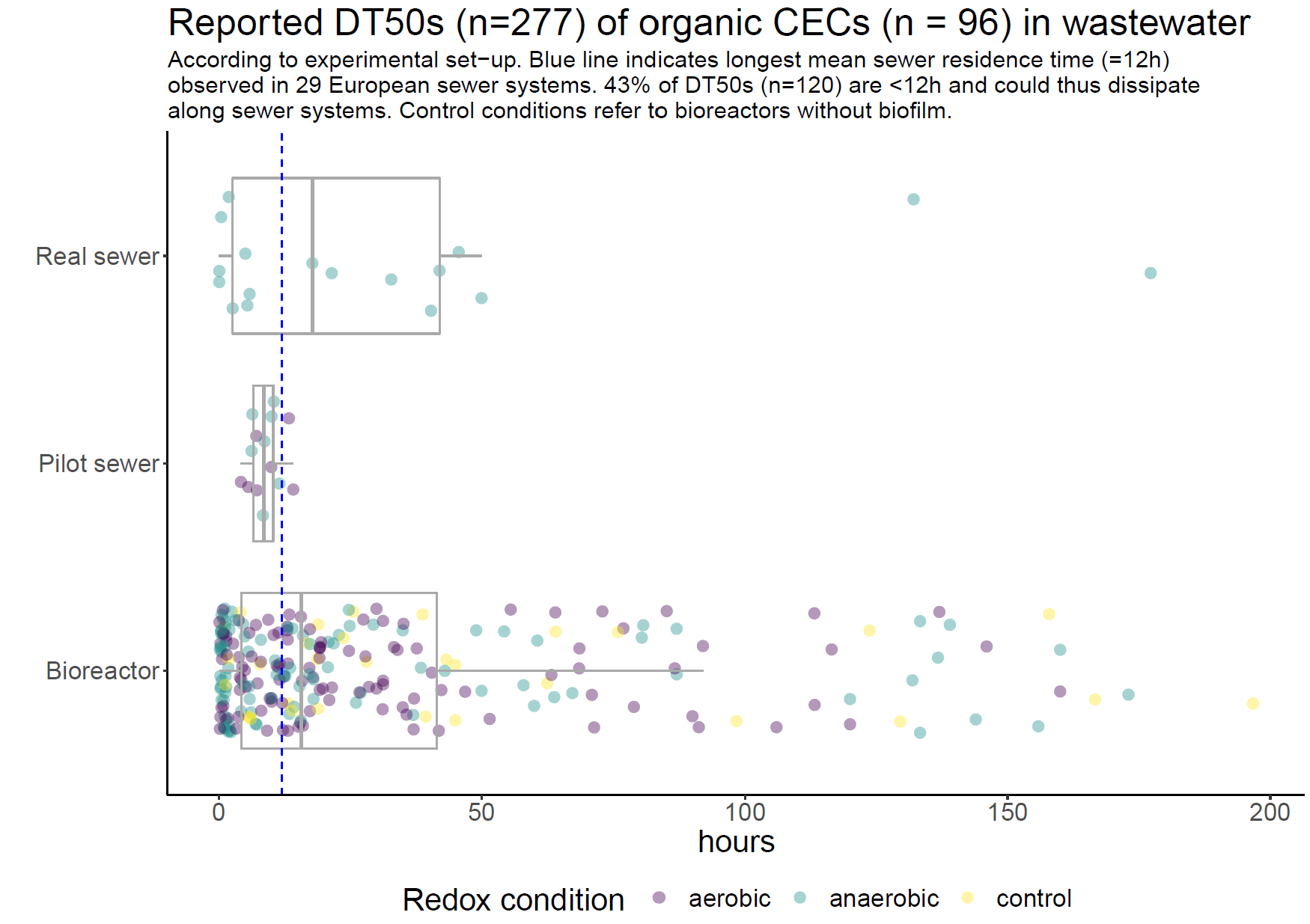


Figure S 7 - Reported DT50s (n=277) 96 CECs according to type of study and prevailing redox conditions. Blue dotted line represents longest mean sewer residence time (=12h) reported for 29 sewer catchments in Europe in Ort et al. 2014. All points left to this line (n=120) indicate compounds for which the load would at least halve upon reaching the WWTP.

1. **Compound-specific data analysis**


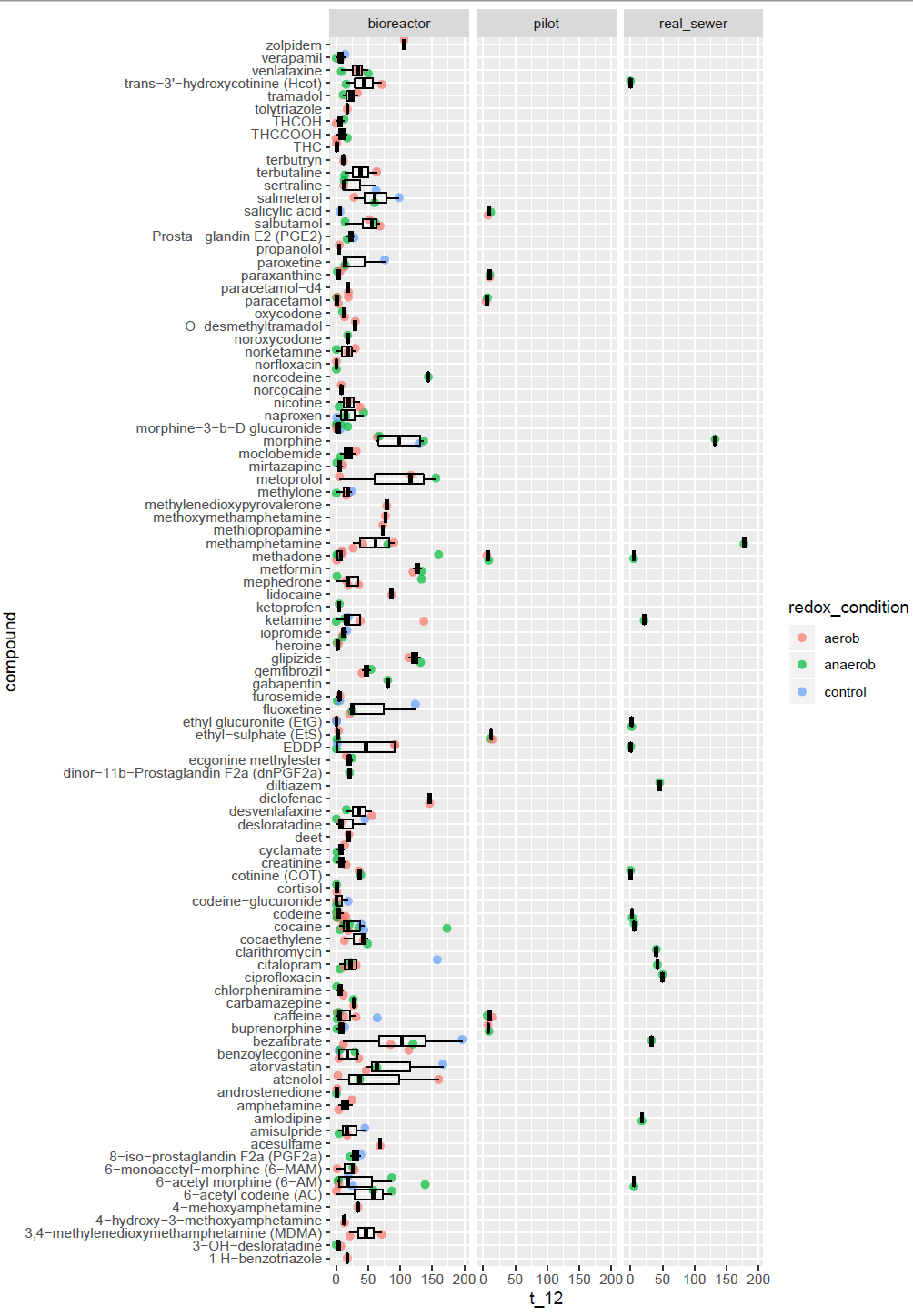


Figure S 8 – Compounds and their observed DT50s per study type and redox conditions. All compounds with at least with at least one half-live per study type and redox condition. Each dot represents an individual half-live. Boxplots for all redox conditions per compound and study type.


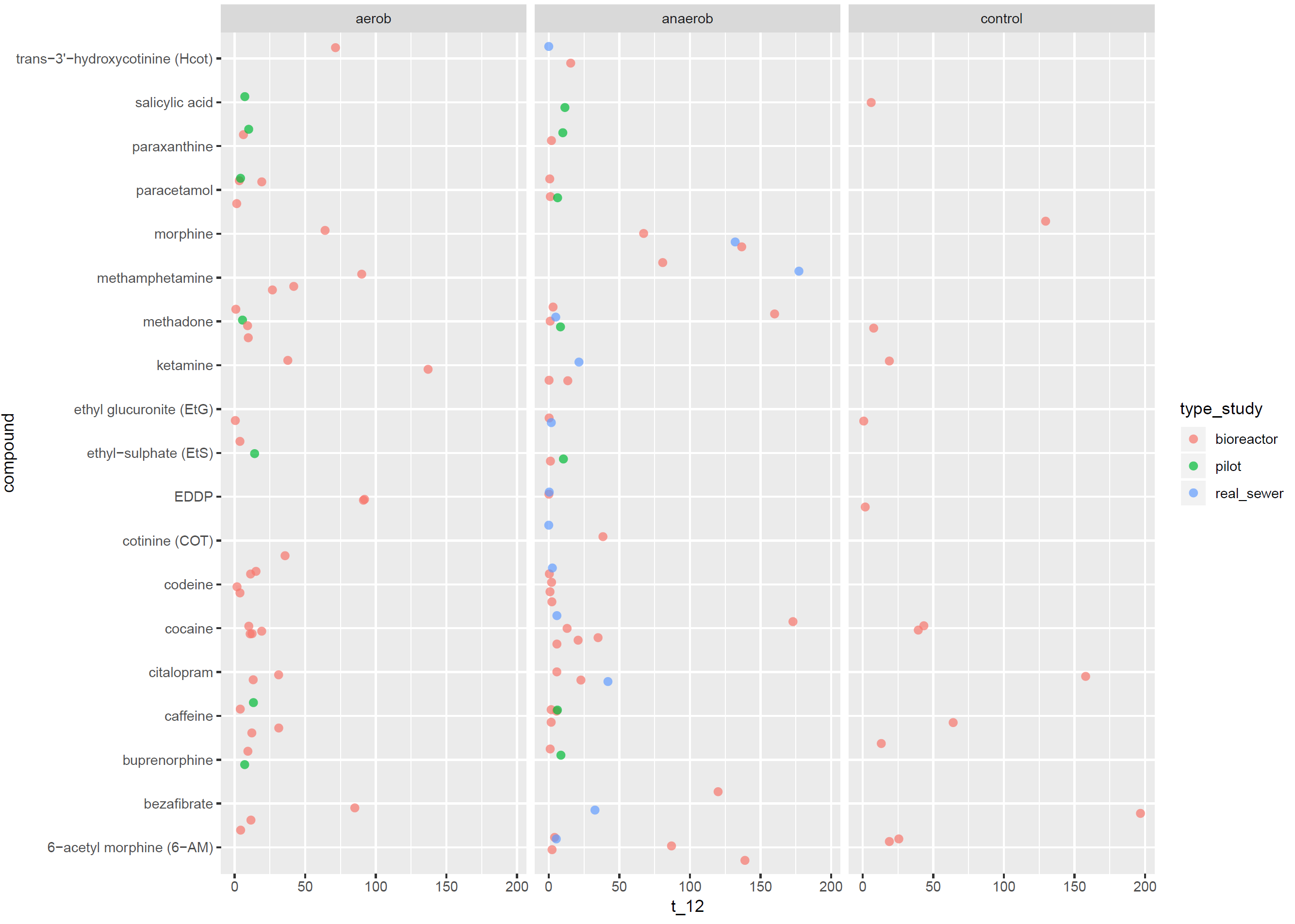


Figure S 9 - Compounds with at least one half-life for 2 different study types per redox condition.

1. **Compare half-lives to BIOWIN and OECD data**

For none of these compounds OECD 314 was published at Web of Science. Only one study published results of OECD 301 D and F for one compound (EtS). EtS remained stable for 28 days under closed bottle (301D) and stable for 6 days under manometric respiratory test (301F).

Compound identifiers (CAS number and canonical SMILES) as well as chemical properties were collected from PubChem (here only experimental data presented). Via CAS and SMILE were introduced to EpiSuite to generate BIOWIN3 (ultimate degradation survey model) and BIOWIN4 (primary biodegradation survey model) estimates. Half-lives based on BIOWIN are here transformed into hour (=BIOWIN t ½ *24h). EDDP was not found in PubChem.

1. **Statistical analysis of bioreactor data**

Data most abundant for bioreactor tests, therefor do statistical analysis for this group. Quantify variability among studies conducted under same circumstances (aerobic, anaerobic bioreactors Australian studies) with variability among studies conducted at different locations ((aerobic, anaerobic bioreactors of non-Australian studies).

The dataset was split into 4 subsets:

1a) Australian bioreactors aerobic conditions

1b) Australian bioreactors anaerobic conditions

2a) Non-Australian bioreactors aerobic conditions

2b) Non-Australian bioreactors anaerobic conditions

Results: Boxplots were created for each subset; for compounds with at least 2 half-lives within subset.

**1a: Australian bioreactors aerobic conditions**


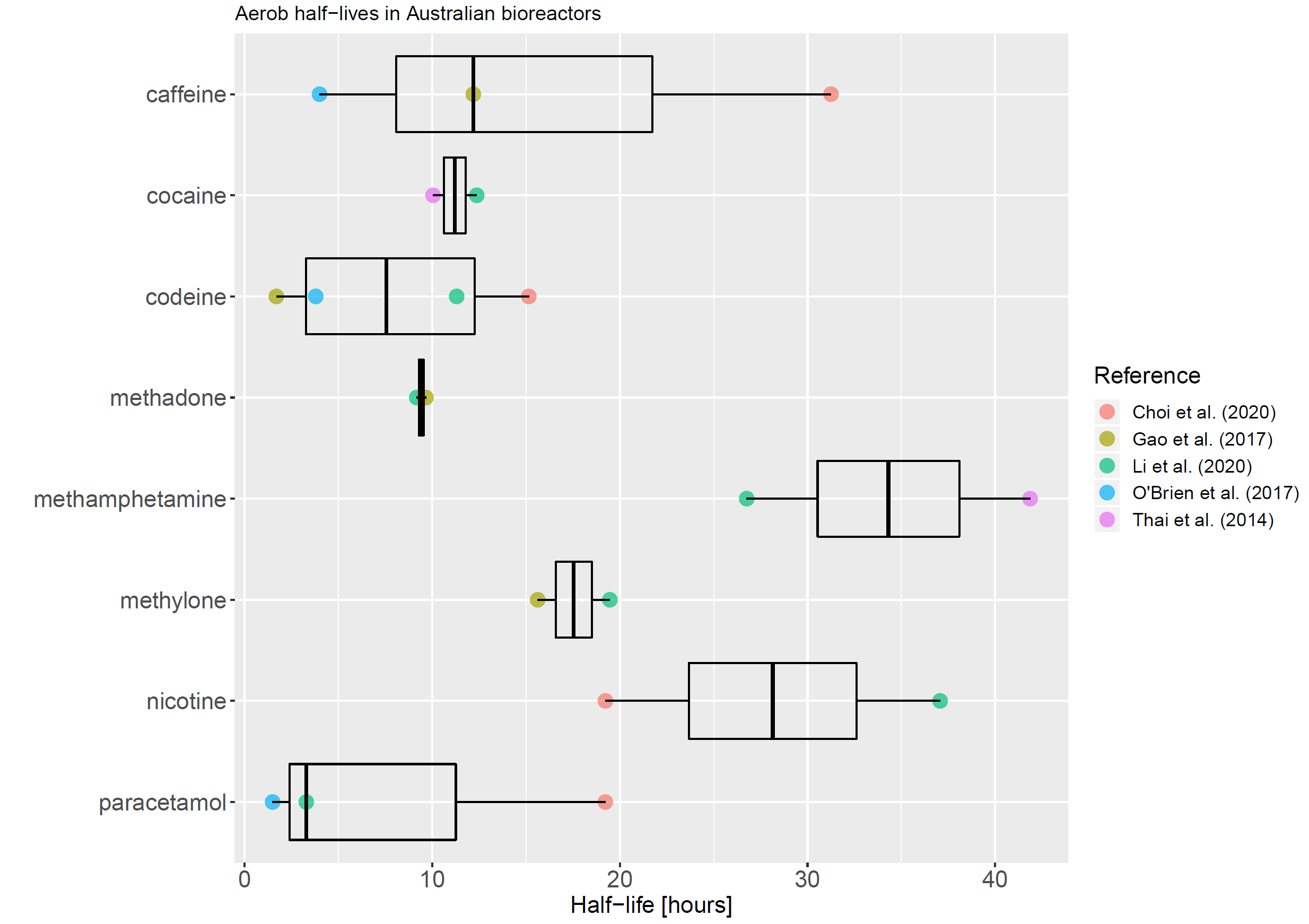


**1b: Australian bioreactors anaerobic conditions**
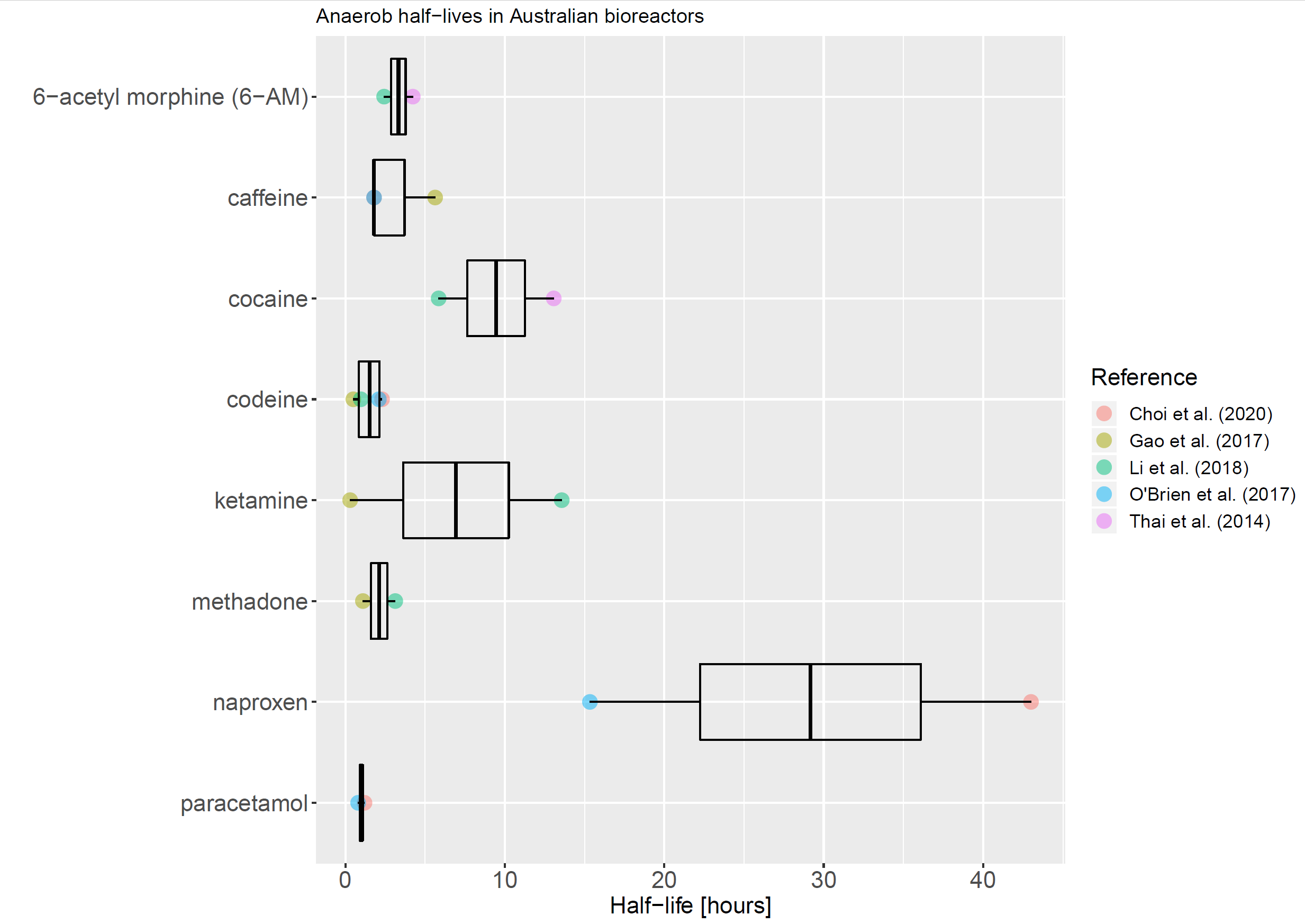


**2a: Non-Australian bioreactors aerobic conditions**
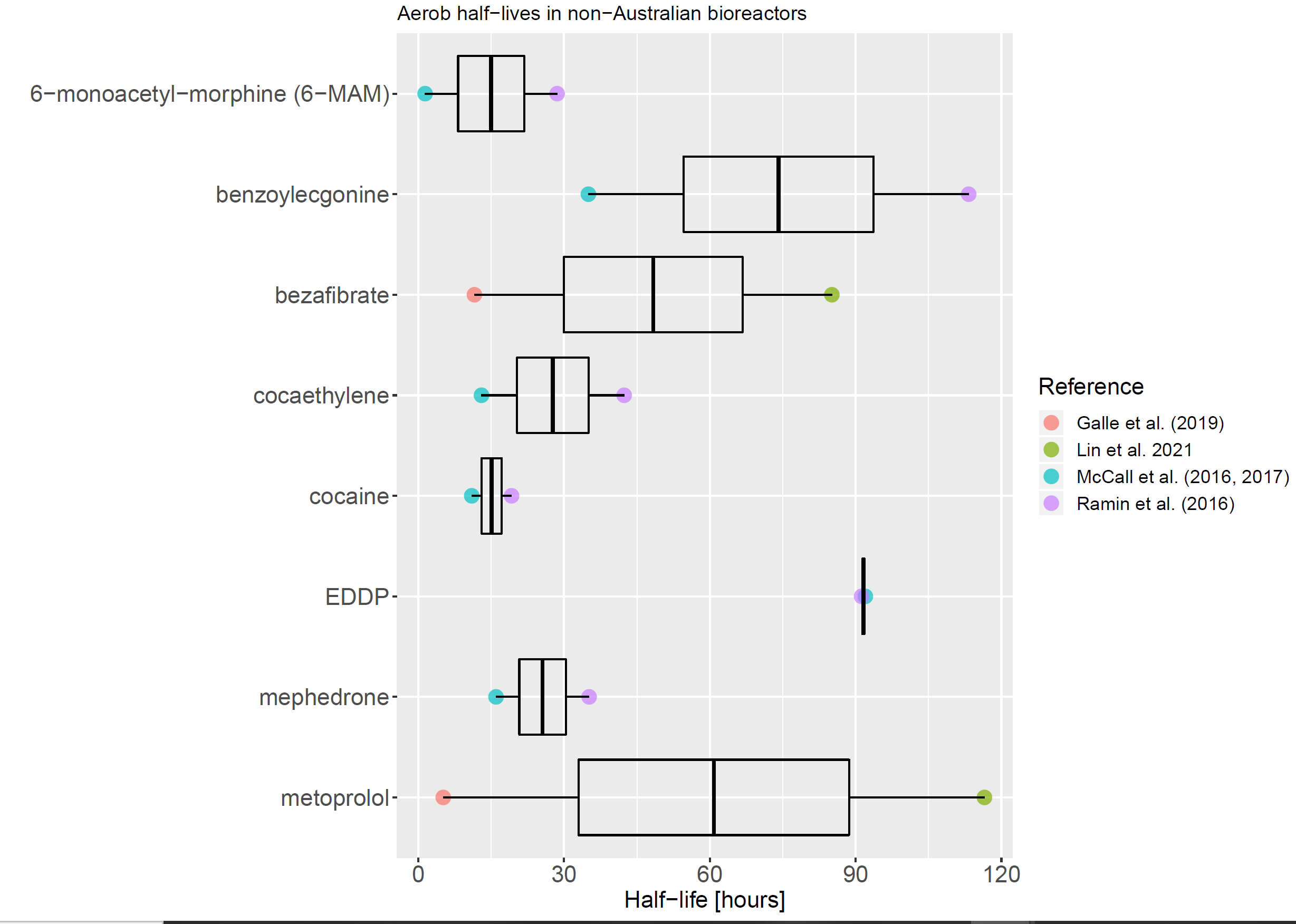


**2b: Non-Australian bioreactors anaerobic conditions**


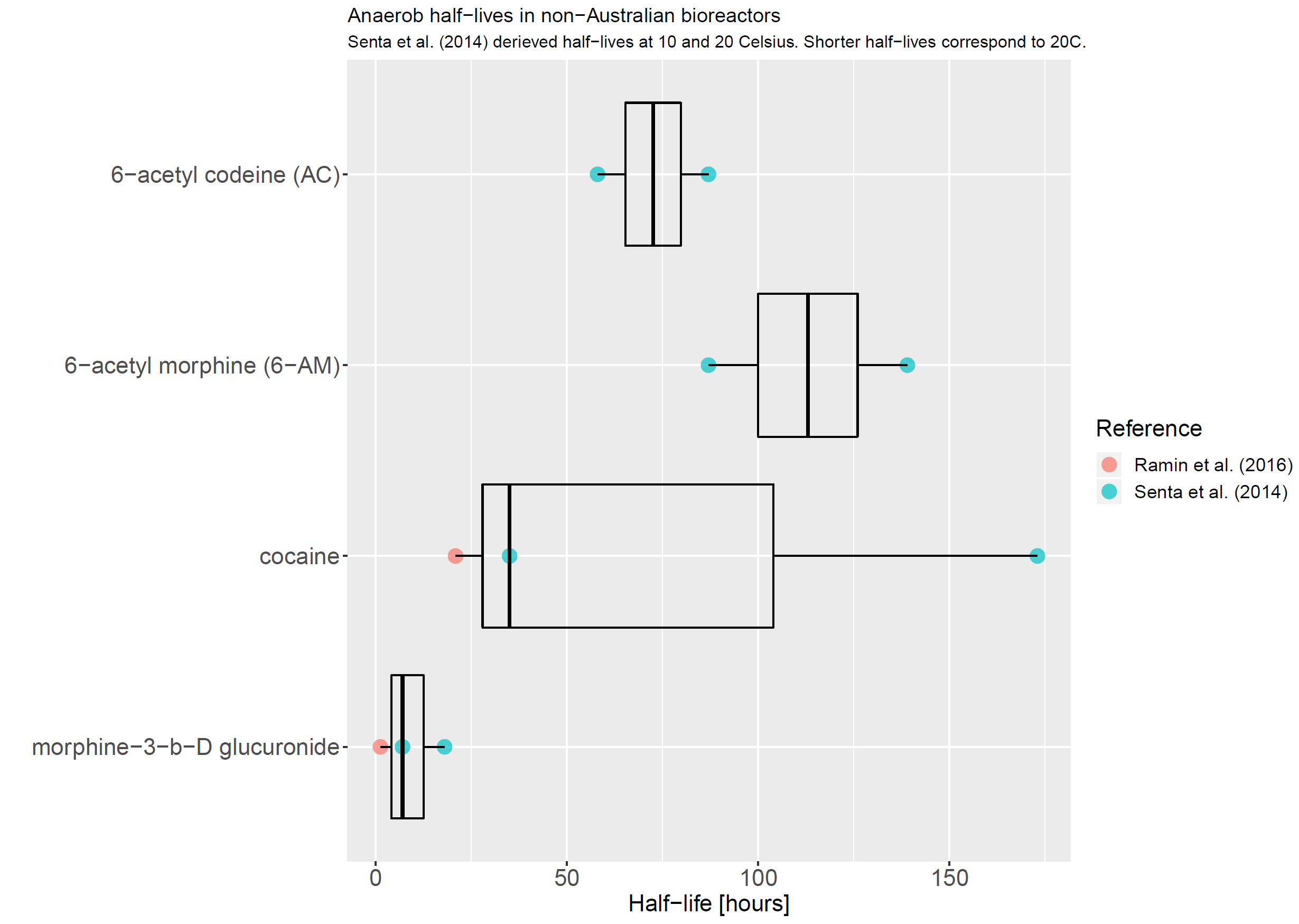


Besides the boxplots, basic statistics were applied to quantify the variability among subsets. Hereto, normal distribution was assumed (based on the boxplots) and the mean, standard deviation, variation (=sd^2^) and the coefficient of variation (= sd/mean) were assessed for each compound with more than one half-life per subset. Number of half-lives in column “n”. Cocaine is the only compound with multiple half-lives within each of the subsets, marked in yellow.

Statistics to 1a:

| **Aerobic bioreactors Australia** | |  |  |  |  |  |
| --- | --- | --- | --- | --- | --- | --- |
|  | compound | t_12.mean | t_12.sd | t_12.var | n | coefficient of variation |
| 1 | caffeine | 15.8 | 14.0 | 195.5 | 3 | 88% |
| 2 | cocaine | 11.2 | 1.6 | 2.7 | 2 | 15% |
| 3 | codeine | 8.0 | 6.3 | 39.8 | 4 | 79% |
| 4 | methadone | 9.4 | 0.3 | 0.1 | 2 | 4% |
| 5 | methamphetamine | 34.3 | 10.7 | 114.0 | 2 | 31% |
| 6 | methylone | 17.5 | 2.7 | 7.4 | 2 | 15% |
| 7 | nicotine | 28.1 | 12.6 | 159.1 | 2 | 45% |
| 8 | paracetamol | 8.0 | 9.8 | 95.3 | 3 | 122% |

Statistics to 2a:

| **Aerobic bioreactors Rest** | |  |  |  |  |  |
| --- | --- | --- | --- | --- | --- | --- |
|  | compound | t_12.mean | t_12.sd | t_12.var | n | coefficient of variation |
| 1 | 6-monoacetyl-morphine(6-MAM) | 15.0 | 19.2 | 369.1 | 2 | 128% |
| 2 | benzoylecgonine | 74.1 | 55.3 | 3062.4 | 2 | 75% |
| 3 | bezafibrate | 48.3 | 52.0 | 2705.1 | 2 | 108% |
| 4 | cocaethylene | 27.7 | 20.8 | 431.1 | 2 | 75% |
| 5 | cocaine | 15.1 | 5.8 | 33.6 | 2 | 38% |
| 6 | EDDP | 91.6 | 0.6 | 0.3 | 2 | 1% |
| 7 | mephedrone | 25.6 | 13.5 | 183.4 | 2 | 53% |
| 8 | metoprolol | 60.8 | 78.8 | 6201.7 | 2 | 129% |

Statistics to 1b:

| **Anaerobic bioreactors Australia** | | |  |  |  |  |
| --- | --- | --- | --- | --- | --- | --- |
|  | compound | t_12.mean | t_12.sd | t_12.var | n | coefficient of variation |
| 1 | 6-acetyl morphine (6-AM) | 3.3 | 1.3 | 1.6 | 2 | 38% |
| 2 | caffeine | 3.1 | 2.2 | 4.9 | 2 | 72% |
| 3 | cocaine | 9.5 | 5.1 | 26.1 | 2 | 54% |
| 4 | codeine | 1.5 | 0.9 | 0.8 | 4 | 59% |
| 5 | ketamine | 6.9 | 9.4 | 88.0 | 2 | 135% |
| 6 | methadone | 2.1 | 1.4 | 2.1 | 2 | 68% |
| 7 | naproxen | 29.2 | 19.6 | 382.6 | 2 | 67% |
| 8 | paracetamol | 1.0 | 0.3 | 0.1 | 2 | 28% |

Statistics to 2b:

| **Anaerobic bioreactors Rest** | |  |  |  |  |  |
| --- | --- | --- | --- | --- | --- | --- |
|  | compound | t_12.mean | t_12.sd | t_12.var | n | coefficient of variation |
| 1 | 6-acetyl codeine (AC) | 72.5 | 20.5 | 420.5 | 2 | 28% |
| 2 | 6-acetyl morphine (6-AM) | 113.0 | 36.8 | 1352.0 | 2 | 33% |
| 3 | cocaine | 76.3 | 84.1 | 7064.6 | 3 | 110% |
| 4 | morphine-3-b-D glucuronide | 8.7 | 8.5 | 72.7 | 3 | 98% |

Note: Data for subset 2b contains for each compound half-lives derived at 4 and 20°C (Senta et al. 2014). Therefore, statistics are not directly comparable to the other subsets.

### PCA and clustering

Using the created dataset, we attempted to find relationships between DT50s and compound properties as well as between DT50s and experimental settings. Hereto, we performed correlation tests, principal component analysis (PCA) and hierarchical clustering (HC). Data analysis was performed in R version 3.6.1. However, except for some weak but significant correlations (Pearson) between aerobic bioreactor DT50s and wastewater temperature (R = 0.29, p < 0.05); DT50s and AV ratio (-0.25, p < 0.05); and geomean DT50 and log K_d_ (R= -0.46, p < 0.05), no meaningful patterns were found. This suggests that our dataset is currently too small in terms of DT50s per unique compound to explain any of the theoretical links between experimental settings or compound properties and observed DT50s. In more general terms, our lack of conclusive results could also indicate that our hypothesized explanatory variables (=compound properties, experimental settings) might not well reflect the intended response variable (= DT50).

### PCA of DT50s and experimental settings


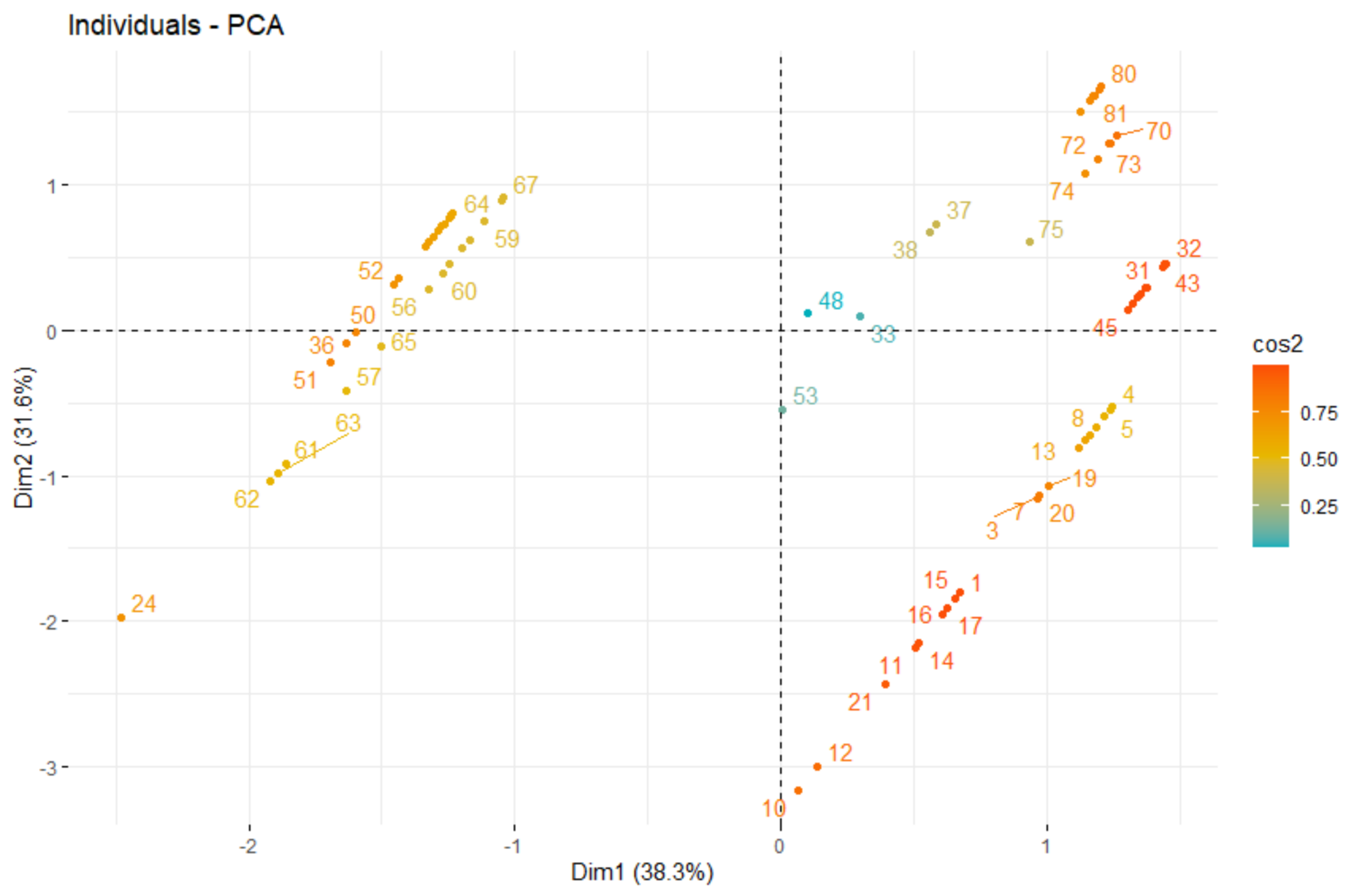

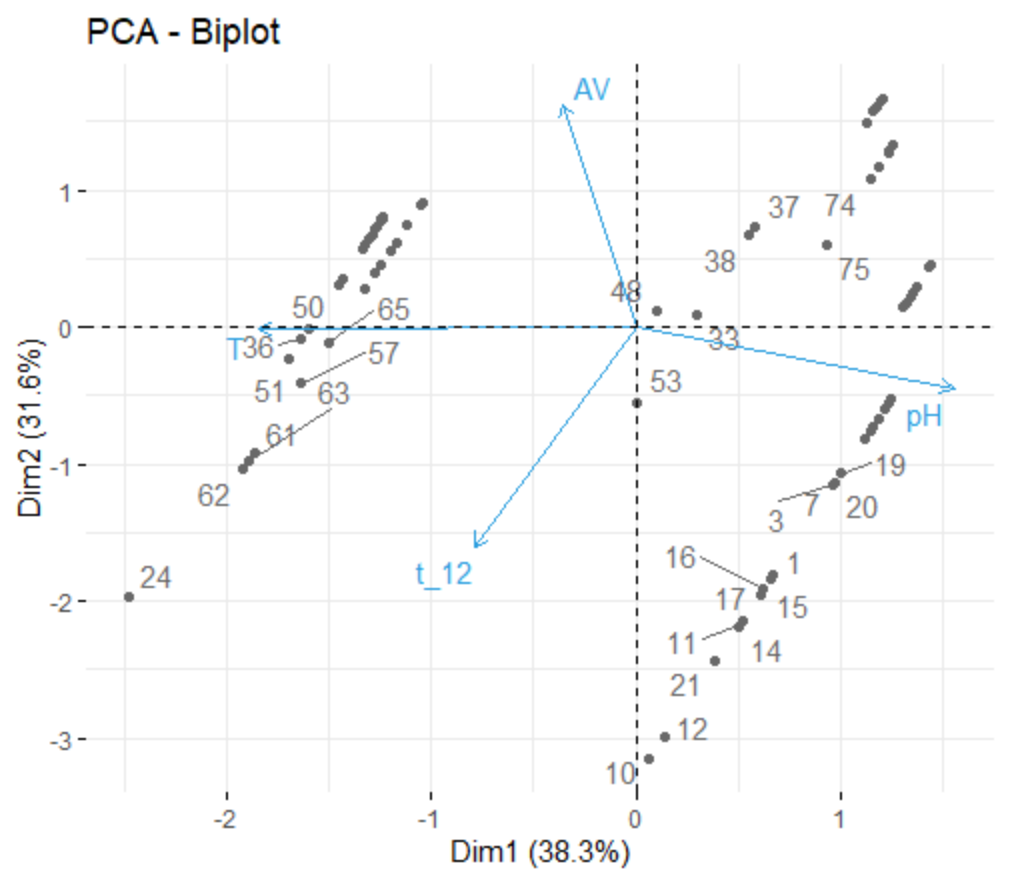


Figure S 10 - PCA of individual DT50s (left) and PCA Biplot (right). Grouping represents studies with similar experimental settings from which DT50s were obtained.


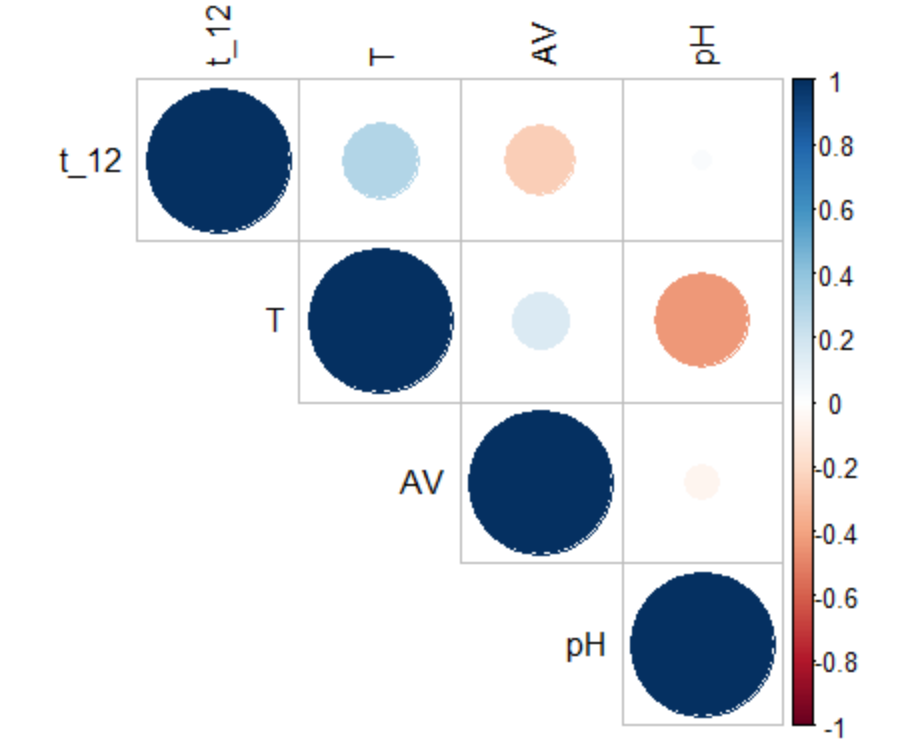


Figure S 11 - Correlation matrix (Pearson) of DT50s and experimental settings. DT50s and wastewater temperature were positively correlated (0.29, p <0.05) while DT50s and AV-ratios were negatively correlated (-0.25, p <0.05). No correlation was observed between DT50s and pH (0.02, p >0.5).

PCA of aggregated DT50s and compound properties

Since the dataset contains multiple DT50s for some compounds, all DT50s were aggregated into the geomean DT50 so to avoid double counting.


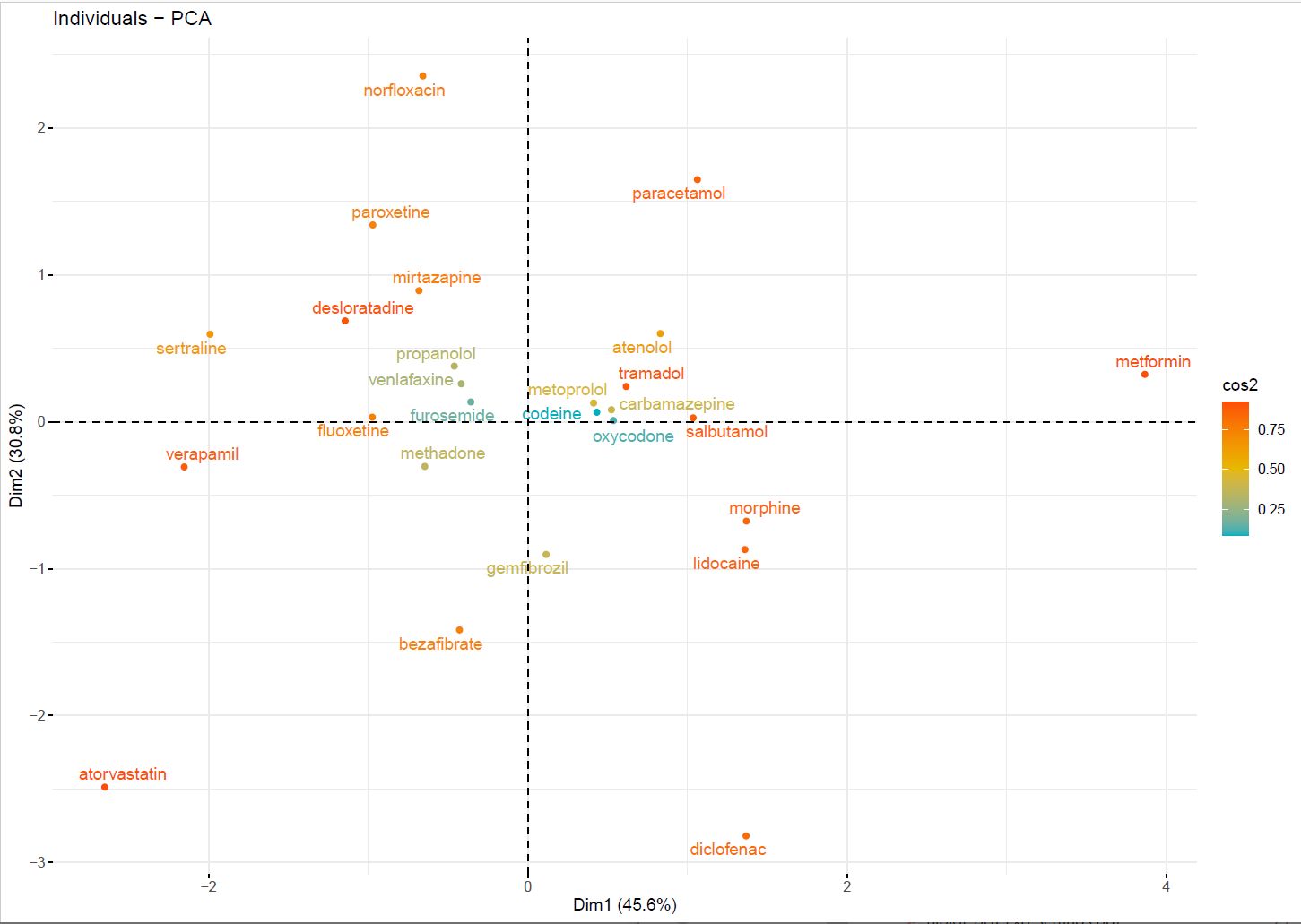

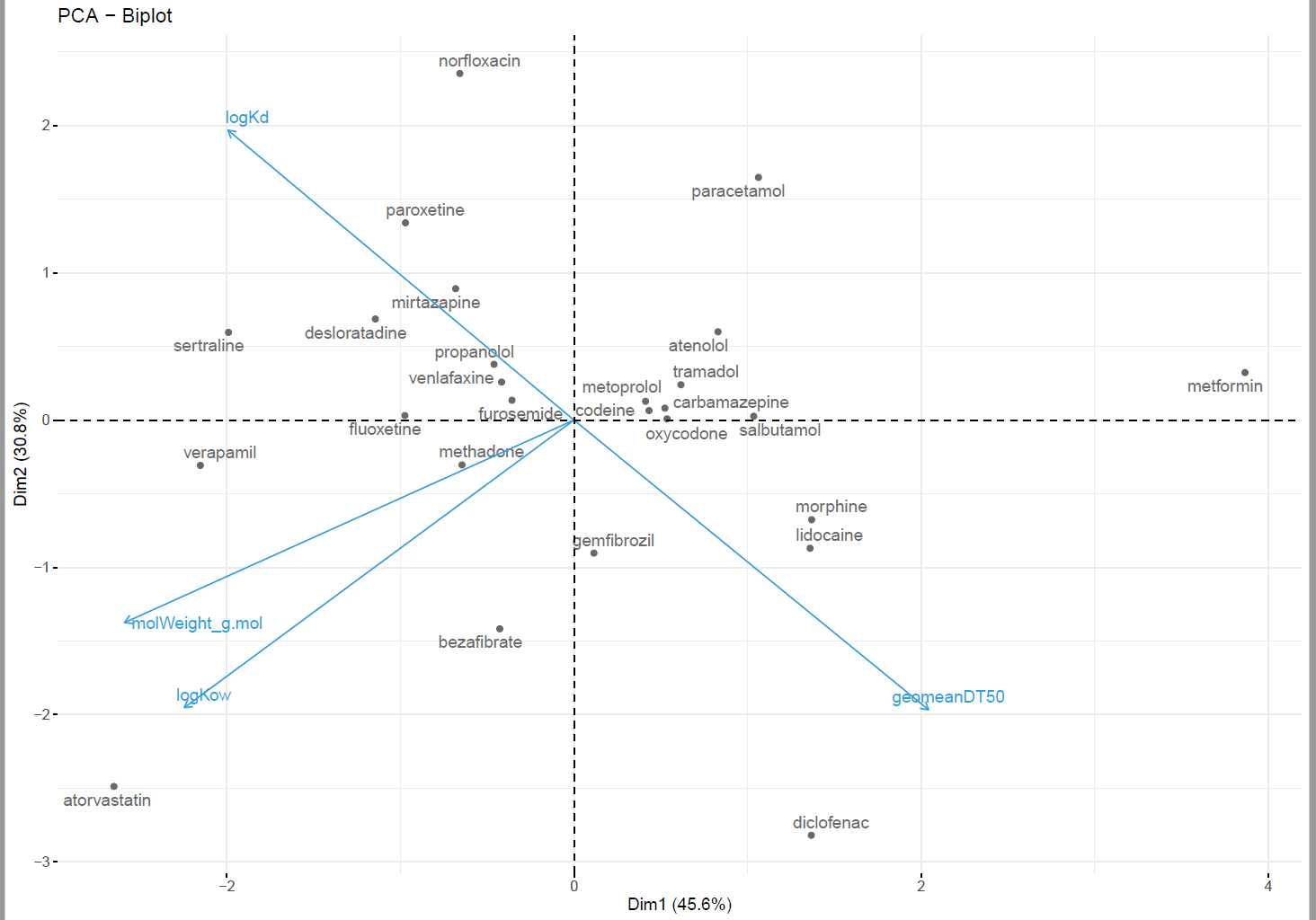


Figure S 12 - PCA plot on individual compounds (upper) and PCA biplot (lower). No obvious groupings were observed.


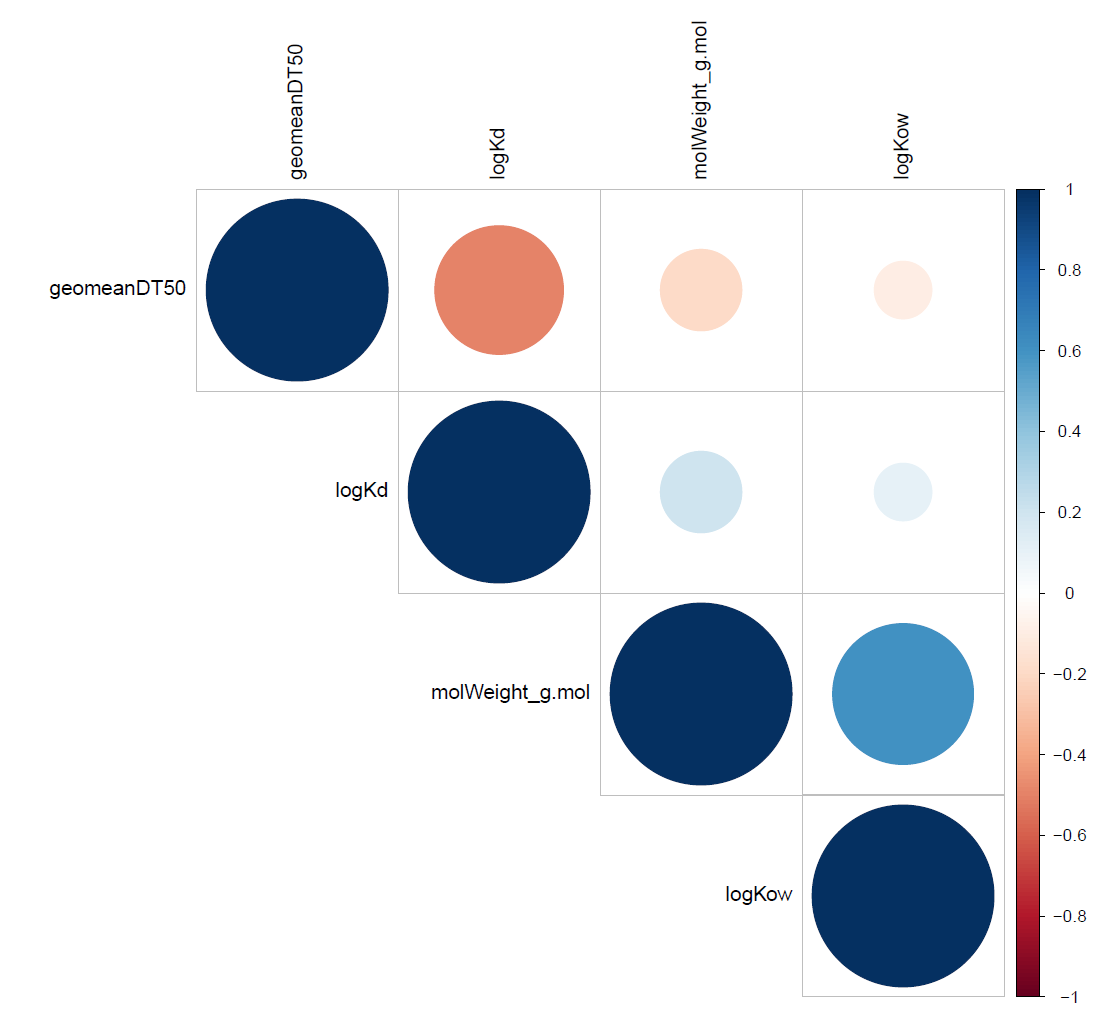


Figure S 13 - Correlation matrix (Pearson) of DT50s aggregated per compound (as geomean) and compound properties. Only log Kd and aggregated DT50s showed a statistically significant correlation (-0.46, p <0.05) meaning that shorter DT50s were related to compounds with higher Kd values. This is counter-intuitive to the traditional approach of sorption as compounds with higher Kd values would sorb and would therefore not be bioavailable. Given that dissipation curves observed for most compounds followed 1^st^-order kinetics indicating that biological processes are main drivers of dissipation, this finding could suggest that compounds in wastewater could first sorb (e.g. to biomass) and then undergo biotransformation.

## Search terms section 5 ‘Integrated fate modelling of CECs in sewers’

'integrated model*' AND (CEC* OR pharmaceutical* OR micropollutant*) AND fate AND (wastewater OR sew* OR urban) (=49 results)

(sewer* OR 'wastewater system*') AND model* AND (fate OR transport) AND (micropol* OR CEC* OR pharmaceutical*) NOT (monit* OR treat*) (=27 results)

## References

Becker, R. A., & Wilks, A. R. (2018a). mapdata: Extra Map Databases.

Becker, R. A., & Wilks, A. R. (2018b). *maps: Draw Geographical Maps*. Retrieved from https://cran.r-project.org/package=maps

Brown, A. K., Ackerman, J., Cicek, N., & Wong, C. S. (2020). In situ kinetics of human pharmaceutical conjugates and the impact of transformation, deconjugation, and sorption on persistence in wastewater batch bioreactors. *Environmental Pollution*, *265*, 114852. https://doi.org/10.1016/j.envpol.2020.114852

Chang, W., & Wickham. (2020). *ggvis: Interactive Grammar of Graphics*. Retrieved from https://cran.r-project.org/package=ggvis

Cormier, G., Barbeau, B., Arp, H. P. H., & Sauvé, S. (2015). The degradation behaviour of nine diverse contaminants in urban surface water and wastewater prior to water treatment. *Environmental Sciences: Processes and Impacts*, *17*(12), 2051–2065. https://doi.org/10.1039/c5em00338e

Gallé, T., Koehler, C., Plattes, M., Pittois, D., Bayerle, M., Carafa, R., … Hansen, J. (2019). Large-scale determination of micropollutant elimination from municipal wastewater by passive sampling gives new insights in governing parameters and degradation patterns. *Water Research*, *160*, 380–393. https://doi.org/10.1016/j.watres.2019.05.009

Harrell, F. E. J., Dupont, C., & ... (2021). *Hmisc: Harrell Miscellaneous*. Retrieved from https://cran.r-project.org/package=Hmisc

McCall, A. K., Palmitessa, R., Blumensaat, F., Morgenroth, E., & Ort, C. (2017). Modeling in-sewer transformations at catchment scale – implications on drug consumption estimates in wastewater-based epidemiology. *Water Research*, *122*, 655–668. https://doi.org/10.1016/j.watres.2017.05.034

McCall, A. K., Scheidegger, A., Madry, M. M., Steuer, A. E., Weissbrodt, D. G., Vanrolleghem, P. A., … Ort, C. (2016). Influence of different sewer biofilms on transformation rates of drugs. *Environmental Science and Technology*, *50*(24), 13351–13360. https://doi.org/10.1021/acs.est.6b04200

McIlroy, D. (2020). *mapproj: Map Projections*. Retrieved from https://cran.r-project.org/package=mapproj

Neuwirth, E. (2014). *RColorBrewer: ColorBrewer Palettes*. Retrieved from https://cran.r-project.org/package=RColorBrewer

Ort, C., van Nuijs, A. L. N., Berset, J. D., Bijlsma, L., Castiglioni, S., Covaci, A., … Thomas, K. V. (2014). Spatial differences and temporal changes in illicit drug use in Europe quantified by wastewater analysis. *Addiction*, *109*(8), 1338–1352. https://doi.org/10.1111/add.12570

R Core Team. (2019). R: A language and environment for statistical computing. Vienna, Austria: R Foundation for Statistical Computing. Retrieved from https://www.r-project.org/

Ramin, P., Brock, A. L., Causanilles, A., Valverde-Pérez, B., Emke, E., De Voogt, P., … Plósz, B. G. (2017). Transformation and Sorption of Illicit Drug Biomarkers in Sewer Biofilms. *Environmental Science and Technology*, *51*(18), 10572–10584. https://doi.org/10.1021/acs.est.6b06277

Ramin, P., Brock, A. L., Polesel, F., Causanilles, A., Emke, E., De Voogt, P., & Plosz, B. G. (2016). Transformation and sorption of illicit drug biomarkers in sewer systems: Understanding the role of suspended solids in raw wastewater. *Environmental Science and Technology*, *50*(24), 13397–13408. https://doi.org/10.1021/acs.est.6b03049

Ren, B., Shi, X., Jin, X., Wang, X. C., & Jin, P. (2021). Comprehensive evaluation of pharmaceuticals and personal care products (PPCPs) in urban sewers: Degradation, intermediate products and environmental risk. *Chemical Engineering Journal*, *404*(September 2020), 127024. https://doi.org/10.1016/j.cej.2020.127024

Shi, X., Sang, L., Wang, X. C., & Jin, P. (2018). Pollutant exchange between sewage and sediment in urban sewer systems. *Chemical Engineering Journal*, *351*(April), 240–247. https://doi.org/10.1016/j.cej.2018.06.096

South, A. (2011). rworldmap: A New R package for Mapping Global Data. *The R Journal*, *3*(1), 35–43. Retrieved from http://journal.r-project.org/archive/2011-1/RJournal_2011-1_South.pdf

Wickham, H. (2016). *ggplot2: Elegant Graphics for Data Analysis*. Springer-Verlag New York. Retrieved from https://ggplot2.tidyverse.org

Wickham, H., Averick, M., Bryan, J., & ... (2019). Welcome to the tidyverse. *Journal of Open Source Software*, *4*(43), 1686. https://doi.org/10.21105/joss.01686

Wickham, H., François, R., Henry, L., & Müller, K. (2021). *dplyr: A Grammar of Data Manipulation*. Retrieved from https://cran.r-project.org/package=dplyr

Wickham, H., & Seidel, D. (2020). *scales: Scale Functions for Visualization*. Retrieved from https://cran.r-project.org/package=scales
